# Comparison of different insulin resistance surrogates to predict hyperuricemia among U.S. non-diabetic adults

**DOI:** 10.3389/fendo.2022.1028167

**Published:** 2022-12-15

**Authors:** Hao Wang, Jia Zhang, Yuzhu Pu, Shengmei Qin, Huan Liu, Yongming Tian, Zhihong Tang

**Affiliations:** ^1^ Department of Critical Care Medicine, West China Hospital of Sichuan University, Chengdu, China; ^2^ Department of Cardiology, Shanghai Seventh People’s Hospital Affiliated to Shanghai University of Traditional Chinese Medicine, Shanghai, China; ^3^ Medicine & Health Science of Huangshang University, Guangzhou, China

**Keywords:** hyperuricemia, insulin resistance surrogates, diabetes, National Health and Nutrition Examination Survey, American

## Abstract

**Purpose:**

Although it has been well-acknowledged that insulin resistance (IR) plays a critical role in the development of hyperuricemia (HU), specific relationship between IR and HU in non-diabetic patients remains rarely studied, and there is still no large-scale research regarding this issue. This study aims to explore the association between triglyceride glucose (TyG), TyG with body mass index (TyG-BMI), the ratio of triglycerides divided by high-density lipoprotein cholesterol (TG/HDL-C), metabolic score for insulin resistance (METS-IR), and the risk of HU in non-diabetic patients in The United States of America.

**Patients and methods:**

Data from the National Health and Nutrition Examination Survey (NHANES) enrolling a representative population aged ≥18-year-old were included to calculate these four indexes. Logistic regression analysis was applied to describe their associations and calculate odds ratios (OR) while the Receiver Operating Characteristic curve was utilized to assess the prediction ability of these four indexes.

**Results:**

A total of 7,743 people (3,806 males and 3,937 females, mean age: 45.17 ± 17.10 years old) were included in this study, among whom 32.18% suffered from HU. After adjustment for sex, age, ethnicity, education background, smoking status, drinking status, systolic blood pressure (SBP), diastolic blood pressure (DBP), metabolic equivalent values (METs), total cholesterol, low-density lipoprotein cholesterol, and estimated glomerular filtration rate, it showed that all four indexes were closely related to HU. Compared with the lowest quartile, OR of the highest quartile of these four indicators for HU were as following respectively: TyG: 5.61 (95% CI: 4.29–7.32); TyG-BMI: 7.15 (95% CI: 5.56–9.20); TG/HDL-C: 4.42 (95% CI: 3.49–5.60); METS-IR: 7.84 (95% CI: 6.07–10.13). TyG, TyG-BMI, TG/HDL-C and METS-IR had moderate discrimination ability for HU, with an AUC value of 0.66 (95% CI: 0.65–0.68), 0.67 (95% CI: 0.65-0.68), 0.68 (95% CI: 0.67-0.69) and 0.68 (95% CI: 0.66–0.69) respectively. Each index showed better prediction ability for HU risk in females than in males.

**Conclusion:**

It was found that the risk of HU was positively associated with the elevation of TyG, TyG-BMI, TG/HDL-C and METS-IR in a large-scale population of U.S., and TyG-BMI and METS-IR have a better ability to identify HU in both genders.

## 1 Introduction

Elevated serum urate (SU) level, known as hyperuricemia (HU), has emerged as a major global public health issue that associated with gout and a wide spectrum of diseases. HU is caused by increased production of uric acid in and/or decreased excretion of uric acid from the body. Epidemiological studies have shown that HU is an independent risk factor for cardiovascular diseases. It is estimated that a quarter of all deaths in developed countries are related to cardiovascular diseases (CVD) (1). In addition, the mortality rate of CVD ranks top among all lethal factors internationally. According to the Global Burden of Diseases report published by the World Health Organization, 17,858,000 people died from cardiovascular diseases (CVD) in 2016, accounting for 31.4% of all deaths (2).

Insulin resistance (IR) refers to a reduced biological effectiveness of insulin on effector organs (3). High glucose levels, as a result of IR, can contribute to obesity, metabolic syndrome, cardiovascular diseases, and other chronic diseases (4). In order to evaluate IR severity, a homeostatic IR assessment model and a quantitative insulin sensitivity index are used, which require insulin measurement or invasive testing, making it not suitable for large-scale epidemiological studies. In this study, as in previous epidemiological studies, non-insulin-based fasting IR indicators, known as surrogates, were used to identify IR levels, including the triglyceride glucose (TyG), TyG with body mass index (TyG-BMI), the ratio of triglycerides divided by high-density lipoprotein cholesterol (TG/HDL-C) and metabolic score for insulin resistance (METS-IR) (5–8).

Although some studies have explored the correlation between IR and HU, studies comparing the prediction ability of different IR indicators in patients with HU remain rare (9, 10). In addition, previous studies mainly focus on the general population including diabetics, ignoring the potential risk of IR in non-diabetic populations with HU (11, 12). The association among TyG, TyG-BMI, TG/HDL-C, METS-IR and HU in non-diabetic patients is still unclear. Therefore, this study will explore the predictive value of TyG, TyG-BMI, TG/HDL-C and METS-IR in non-diabetic patients with HU, identifying an optimal predictor of HU.

## 2 Material and methods

### 2.1 Study population

**Figure 1:**
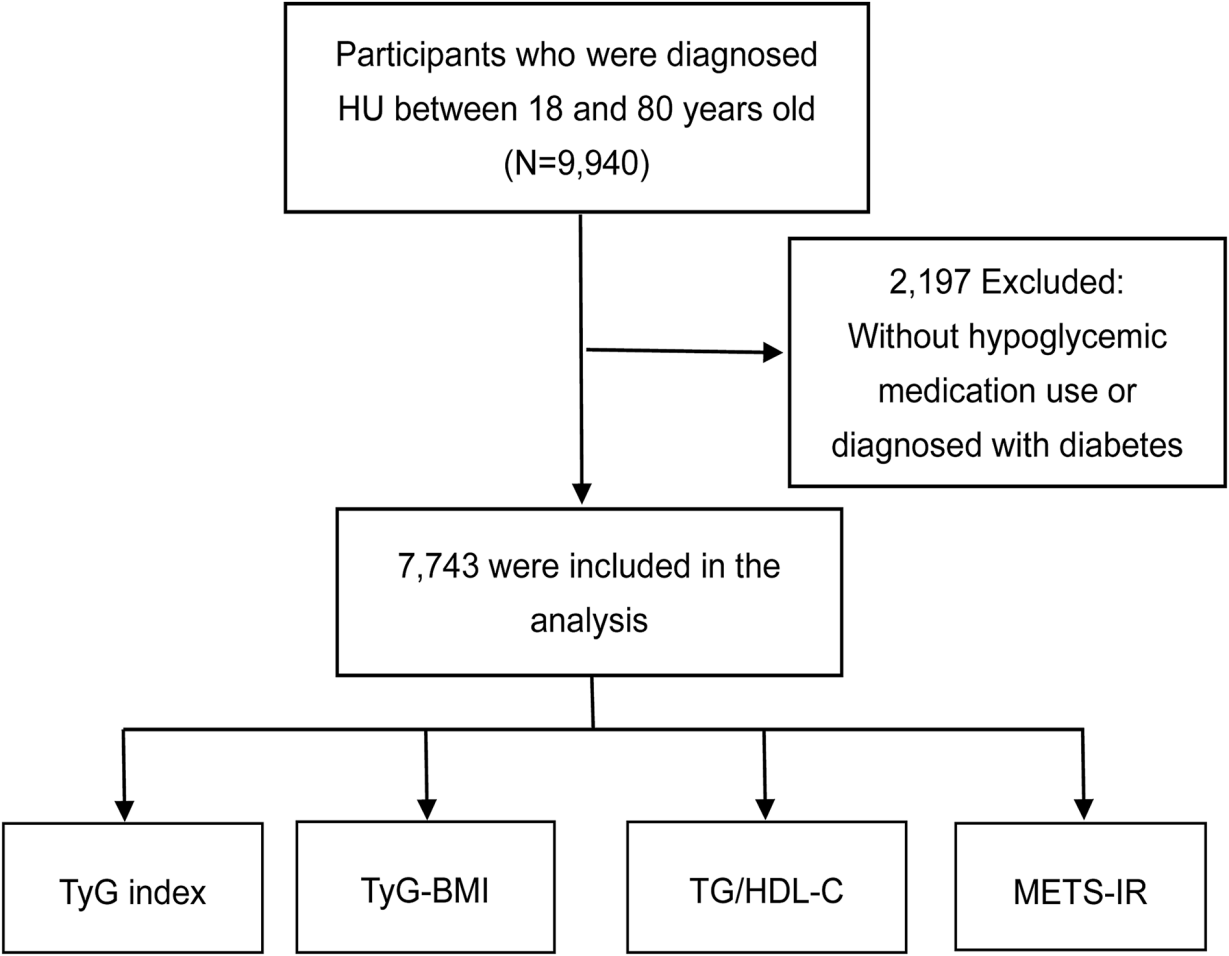
Flowchart of the study. HU, hyperuricemia; TyG, triglyceride glucose; TyG-BMI, triglyceride glucose with body mass index; TG/HDL-C, the ratio of triglycerides divided by high-density lipoprotein cholesterol; METS-IR, metabolic score for insulin resistance.

NHANES is a cross-sectional survey designed to assess the health and nutritional status of, non-institutionalized population in the United States. The survey adopted a complex, stratified, multistage, and probability-cluster sampling design pattern. All of the datasets were downloaded and analyzed directly (http://www.cdc.gov/nchs/nhanes/htm). Data of NHANES 2011–2018 cycle was selected. All 9,940 individuals were above 18 years old (18-80 years old), and had integrate data sets of uric acid (UA), fasting glucose (FPG), total cholesterol (TC), body mass index (BMI), high-density lipoprotein cholesterol (HDL-C) and total triglyceride (TG). Among them, 2,197 participants were excluded for information lack of “hypoglycemic medication” and “diabetes diagnosis”, thus 7,743 patients were enrolled into the final analysis. **Figure 1** is a flowchart of participant enrollment.

### 2.2 Definitions of TyG, TyG-BMI, TG/HDL-C and METS-IR score

The non-insulin-based IR indices of TyG, TG/HDL-C and METS-IR were calculated by the following equations: TyG = ln [(TG (mg/dL) × FPG (mg/dL)/2]; TyG-BMI=TyG ×BMI; TG/HDL-C = TG (mg/dL)/HDL-C (mg/dL); METS-IR = ln [(2 * FPG (mg/dL)) + TG (mg/dL)] * BMI/ln (HDL-C (mg/dL)) (5–8).

### 2.3 Serum uric acid measurement

The main indicator of this study was HU. Use Beckman UniCel^®^ DxC800 Synchron or Beckman Synchron LX20 (Beckman Coulter, Inc., Brea, CA, USA) to detect serum uric acid levels through oxidizing uric acid to form allantoin and H_2_O_2_. HU was defined as those with a UA level ≥6.0 mg/dL (13).

### 2.4 The diagnosis of diabetes

Diabetes was diagnosed when patients met one or more following criteria: (1) patients reporting a diagnosis of diabetes by their doctors (“doctor told you have diabetes”); (2) glycohemoglobin (HbA1c)>6.5%; (3) fasting blood glucose≥7.0mmol/l; (4) random blood glucose≥11.1mmol/l; (5) oral glucose tolerance test (OGTT) two-hour blood glucose≥11.1 mmol/L.

### 2.5 Covariates

Covariates were chosen based on the literature and conceptual significance (9, 11, 12). Covariates included gender, age, ethnicity, education level, smoking, drinking, systolic blood pressure (SBP), diastolic blood pressure (DBP), metabolic equivalent value (MET), total cholesterol (TC), low-density lipoprotein cholesterol (LDL-C) and estimated glomerular filtration rate (eGFR). Among them, gender, age and ethnicity were derived from NHANES interviews. Educational level was divided into three categories: less than high school, high school, and more than high school. Smoking status was categorized into three types: never (no more than 100 cigarettes in lifetime), former (more than 100 cigarettes in lifetime and had quit smoking up to the survey), and current (more than 100 cigarettes in lifetime and is still smoking every several days at least). Drinking status was defined based on self-reports to the question: “In the past 12 months, on those days that you drank alcoholic beverages, on the average, how many drinks did you have?” Blood pressure (systolic and diastolic) were measured in the mobile examination centers using standardized techniques. The Global Physical Activity Questionnaire was used in NHANES to measure physical activity. Participants reported how many days per week and minutes per day they engaged in moderate-intensity physical activity. We calculated total MET-minutes by multiplying the total number of minutes spent doing various activities per week by the metabolic equivalents estimated from the Compendium of Physical Activities. Hyperlipidemia was defined as TG≥150 mg/dl, hypercholesterolemia or lipid-lowering medication. Individuals who met at least one of the following criteria were defined as the hypercholesterolemia: (1) TC ≥200mg/dL; (2) LDL-C ≥130mg/dL; (3) HDL-C <40mg/dL for males; <50mg/dL for females. Hypertension was defined as blood pressure ≥140/90mmHg, a record of a diagnosis of hypertension, or prescription of antihypertensive drugs in the health questionnaires. NHANES datasets also provided laboratory results of TC, LDL-C and serum creatinine. eGFR was estimated by CKD-EPI creatinine equation (14).

### 2.6 Statistical analysis

According to NHANES analytic guidelines, sample weights were incorporated into all analyses for the complexity of survey design (15). The sampling weight was calculated by following formula: fasting sub-sample 10-year mobile examination center (MEC) weight = fasting sub-sample 2-year MEC weight/4. Continuous data are reported as mean ± standard error if normally distributed and as median and interquartile range (IQR) for non-normally distributed data. Categorical variables are presented as numbers in percentage. The Student’s t-test (normal continuous data) or Kruskal Wallis test (non-normal continuous data) were used for comparisons between HU group and non-HU group. Differences in categorical variables were analyzed *via* the chi-square test. Pooled odds ratios (ORs) with 95% confidence intervals (CIs) were calculated to assess the association between four IR surrogates and HU. Area under Receiver Operating Characteristic curve (ROC) was adopted to measure the discrimination ability of different IR surrogates for HU. The cut-off value for the indices was determined by the highest Youden index in the ROC curves. In addition, in the analysis of AUC, we also performed Bootstrap resampling (times = 500) as a sensitivity analysis to verify the stability of the results, and the programming language of construction was shown in **Supplementary material - Methods**. In this study, the R packages “doBy”, “stringr” and “CBCgrps” were used for descriptive statistics; “survival” was used for logistic regression and ORs calculation; “plotrix” and “pROC” were used for plotting (16–21). All statistical analyses were carried out with the statistical software R (http://www.R-project.org, The R Foundation) and EmpowerStats software (http://www.empowerstats.com, X&Y Solutions, Inc., Boston, MA). P-value<0.05 (two-sided) was considered as statistically significant.

## 3 Results

### 3.1 Baseline characteristics

Baseline characteristics are shown in **Table 1**. A total of 7743 people (3806 males and 3937 females, mean age: 45.17 ± 17.10 years old) were included in the study, among whom the prevalence of HU was 32.18%. Participants with HU tended to be older (mean age: 45.81 ± 17.27 years old) than those without HU (mean age: 44.86 ± 17.01 years old), and HU was more common in males (76.61%) than in females (23.39%). Besides, most of HU patients were non-Hispanic White individuals (67.90%). The HU group had lower values of HDL-C and eGFR, and higher values of BMI, TC, TG, LDL-C, FPG, UA, SBP and DBP than non-HU group. TyG, TyG-BMI, TG/HDL-C and METS-IR of the HU group were higher than non-HU one, and the difference was statistically significant (P<0.05).

**Table 1:** Baseline characteristics of subjects.

Variables	Total n=7743	Without HU n=5251	With HU n=2492	P-value
Age (years)	45.17 ± 17.10	44.86 ± 17.01	45.81 ± 17.27	0.02
Gender (n, %)				<0.01
Male	3806 (49.15%)	1890 (35.99%)	1909 (76.61%)	
Female	3937 (50.85%)	3361 (64.01%)	583 (23.39%)	
Ethnicity (n, %)				0.39
Non-Hispanic White	5156 (66.59%)	3463 (65.95%)	1692 (67.90%)	
Non-Hispanic Black	762 (9.84%)	524 (9.98%)	238 (9.55%)	
Mexican American	662 (8.55%)	457 (8.70%)	205 (8.23%)	
Others	1163 (15.02%)	807 (15.37%)	357 (14.33%)	
Education (n, %)				0.01
Less than high school	336 (4.34%)	239 (4.55%)	97 (3.89%)	
High school	2543 (32.84%)	1669 (31.78%)	873 (35.03%)	
More than high school	4864 (62.82%)	3343 (63.66%)	1522 (61.08%)	
BMI (kg/m^2^)	28.43 ± 6.71	27.30 ± 6.14	30.79 ± 7.21	<0.01
Smoking (n, %)				<0.01
Never	4448 (57.45%)	3142 (59.84%)	1306 (52.42%)	
Former	1805 (23.31%)	1085 (20.67%)	719 (28.85%)	
Now	1490 (19.24%)	1024 (19.50%)	467 (18.74%)	
Drinking	2.68 ± 2.37	2.47 ± 2.14	3.09 ± 2.74	<0.01
MET (ml/kg/min)	2400.00 (880.00-6200.00)	2340.00 (840.00-5760.00)	2640.00 (960.00-7200.00)	<0.01
TC (mg/dl)	190.84 ± 39.58	189.27 ± 39.50	194.13 ± 39.54	<0.01
TG (mg/dl)	89.00 (61.00-130.00)	80.00 (57.00-115.00)	108.00 (77.00-162.00)	<0.01
LDL-C (mg/dl)	113.49 ± 34.15	111.62 ± 34.06	117.46 ± 34.00	<0.01
HDL-C (mg/dl)	55.43 ± 16.36	58.34 ± 16.38	49.36 ± 14.54	<0.01
FPG (mg/dl)	99.00 ± 9.72	97.83 ± 9.31	101.43 ± 10.09	<0.01
eGFR (ml/min/1.73 m^2^)	97.21 ± 21.08	99.60 ± 20.06	92.23 ± 22.26	<0.01
UA (mg/dl)	5.30 (4.40-6.30)	4.70 (4.10-5.30)	6.80 (6.30-7.40)	<0.01
SBP (mmHg)	120.21 ± 16.29	118.56 ± 15.75	123.67 ± 16.85	<0.01
DBP (mmHg)	70.16 ± 11.17	69.29 ± 10.73	71.97 ± 11.85	<0.01
TyG	8.42 ± 0.60	8.31 ± 0.56	8.67 ± 0.60	<0.01
TyG-BMI	240.65 ± 63.66	227.61 ± 56.91	267.88 ± 68.25	<0.01
TG/HDL-C	2.39 ± 2.70	1.95 ± 1.98	3.31 ± 3.62	<0.01
METS-IR	41.39 ± 11.88	38.79 ± 10.40	46.82 ± 12.91	<0.01
Hyperlipidemia (n, %)	5052 (65.25%)	3210 (61.13%)	1840 (73.84%)	<0.01
Hypertension (n, %)	2521 (32.56%)	1463 (27.86%)	1056 (42.38%)	<0.01
Lipid lowering medications (n, %)	1093 (14.12%)	679(12.93%)	413 (16.57%)	<0.01
Antihypertensive medications (n, %)	289 (3.73%)	156 (2.97%)	132 (5.30%)	<0.01

HU, hyperuricemia; BMI, body mass index body mass index; MET, metabolic equivalent value; TC, total cholesterol; TG, total triglyceride; LDL-C, low-density lipoprotein cholesterol; HDL-C, high-density lipoprotein cholesterol; FPG, fasting glucose; eGFR, estimated glomerular filtration rate; UA, uric acid; SBP, systolic blood pressure; DBP, diastolic blood pressure; TyG, triglyceride glucose; TyG-BMI, triglyceride glucose with body mass index; TG/HDL-C, the ratio of triglycerides divided by high-density lipoprotein cholesterol; METS-IR, metabolic score for insulin resistance.

### 3.2 Association between four IR surrogates and HU risk

**Table 2** displays the effect sizes of the association between the four IR surrogates quartiles and HU. In the unadjusted model, we observed a positive correlation between four IR surrogates and HU. After adjustment for gender, age, ethnicity, education background, smoking, drinking, SBP, DBP and MET, results showed that the 4th quartile of TyG, TyG-BMI, TG/HDL-C and METS-IR had 4.87-, 6.99-, 4.58- and 6.70-fold HU risk than those in the 1st quartile (Model 2). Similarly, in fully adjusted models, four IR surrogates all had significant ORs for the presence of HU (*p*<0.05) (Model 3).

**Table 2:** ORs and 95% CIs for highest versus the lowest quartiles in logistic regressions predicting presence of HU.

Variables	Model 1	Model 2	Model 3
TyG			
Q2	2.02 (1.75, 2.32)	1.93 (1.56, 2.38)	1.88 (1.51, 2.33)
Q3	2.87 (2.49, 3.30)	3.04 (2.45, 3.78)	3.02 (2.41, 3.79)
Q4	5.63 (4.84, 6.56)	4.87 (3.84, 6.17)	5.61 (4.29, 7.32)
TyG-BMI			
Q2	2.16 (1.87, 2.49	2.07 (1.67, 2.56)	1.99 (1.60, 2.48)
Q3	3.18 (2.76, 3.67)	3.15 (2.53, 3.94)	2.96 (2.35, 3.73)
Q4	4.93 (4.25, 5.72)	6.99 (5.49, 8.89)	7.15 (5.56, 9.20)
TG/HDL-C			
Q2	1.74 (1.50, 2.02)	1.62 (1.30, 2.01)	1.56 (1.24, 1.95)
Q3	2.88 (2.49, 3.33)	2.59 (2.09, 3.22)	2.52 (2.01, 3.17)
Q4	5.76 (4.97, 6.67)	4.58 (3.66, 5.73)	4.42 (3.49, 5.60)
METS-IR			
Q2	2.21 (1.91, 2.55)	1.99 (1.61, 2.47)	2.06 (1.65, 2.58)
Q3	3.31 (2.86, 3.83)	2.90 (2.33, 3.61)	3.07 (2.44, 3.88)
Q4	5.65 (4.86, 6.56)	6.70 (5.29, 8.47)	7.84 (6.07, 10.13)

**Notes:** Values are odds ratio (95%CI) derived from multivariable logistic regression models.

Model 1: unadjusted.

Model 2: adjusted for gender, age, ethnicity, education, smoking, drinking, SBP, DBP and MET.

Model 3: adjusted for all variables in model 2 and TC, LDL-C and eGFR.

TyG, triglyceride glucose; TyG-BMI, triglyceride glucose with body mass index; TG/HDL-C, the ratio of triglycerides divided by high-density lipoprotein cholesterol; METS-IR, metabolic score for insulin resistance; MET, metabolic equivalent value; TC, total cholesterol; LDL-C, low-density lipoprotein cholesterol; eGFR, estimated glomerular filtration rate.

### 3.3 AUCs and cut-off values of four IR surrogates for HU prediction

**Figure 2:**
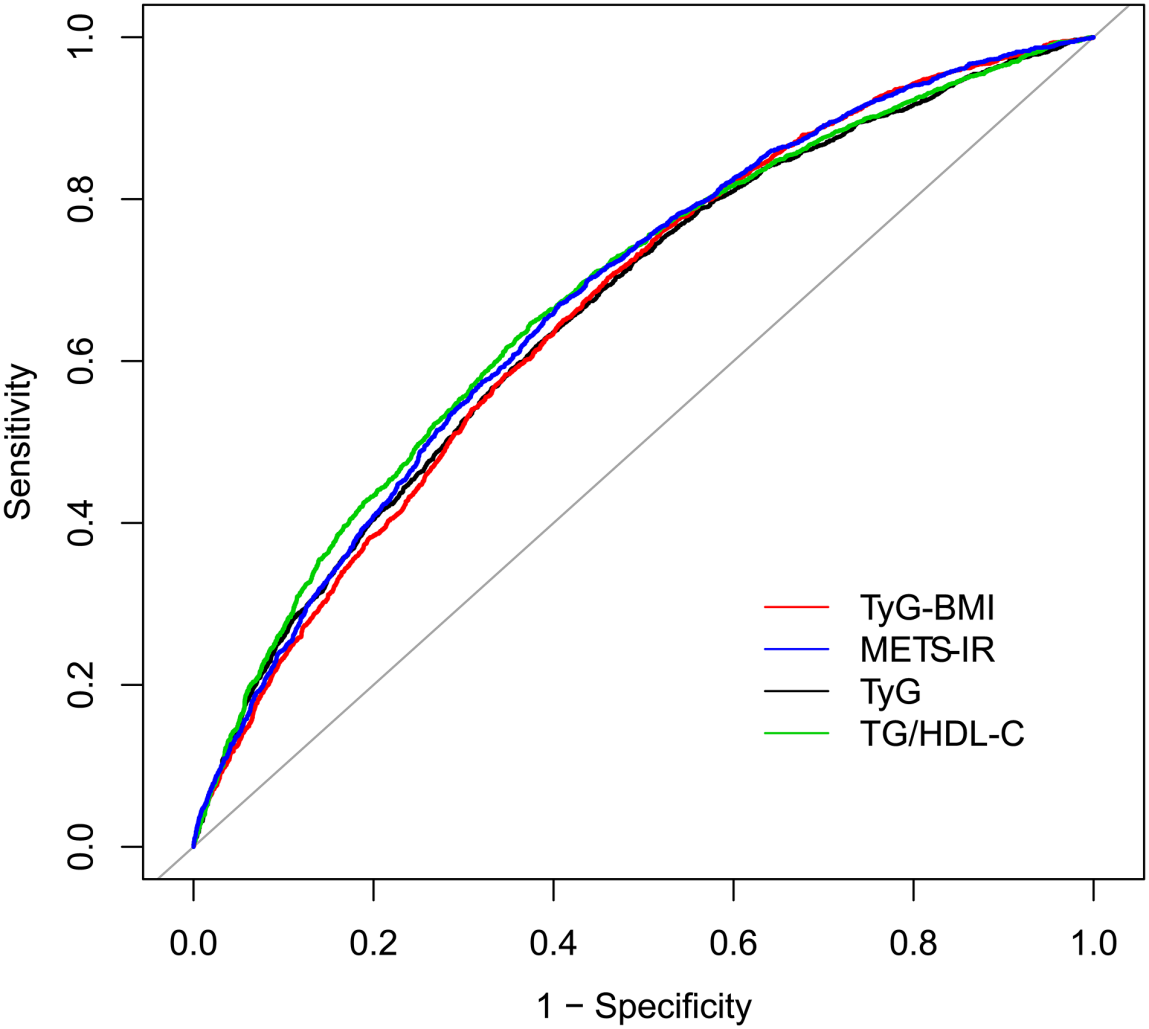
ROC for different IR surrogates to predict HU. HU, hyperuricemia; TyG, triglyceride glucose; TyG-BMI, triglyceride glucose with body mass index; TG/HDL-C, the ratio of triglycerides divided by high-density lipoprotein cholesterol; METS-IR, metabolic score for insulin resistance.

**Figure 3:**
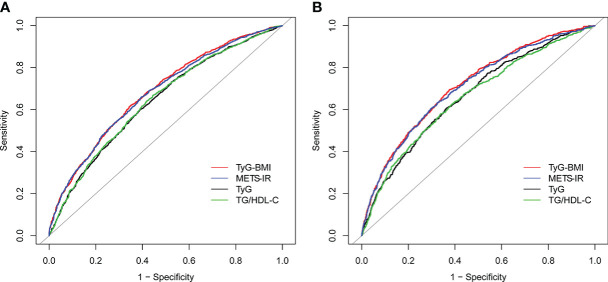
ROC for different IR surrogates to predict HU in **(A)** males; and **(B)** females. HU, hyperuricemia; TyG, triglyceride glucose; TyG-BMI, triglyceride glucose with body mass index; TG/HDL-C, the ratio of triglycerides divided by high-density lipoprotein cholesterol; METS-IR, metabolic score for insulin resistance.

The AUC values of TyG, TyG-BMI, TG/HDL-C and METS-IR to discriminate HU are shown in **Table 3**, **Figures 2**, **3**. TG/HDL-C and METS-IR had higher AUC of 0.68, followed by TyG-BMI (AUC=0.67), TyG (AUC=0.66). The optimal cut-off value of TG/HDL-C and METS-IR based on the specificity and sensitivity was 1.77 and 39.52. Both TyG-BMI and METS-IR showed higher accuracy (AUC=0.68) than TyG and TG/HDL-C (AUC=0.64) in HU prediction of males (**Figure 3A**). The optimal cut-off value of TyG-BMI and METS-IR were 231.26 and 39.52, respectively. Similarly, the AUC value of TyG-BMI and METS-IR are the highest in females (**Figure 3B**). In combination, four IR surrogates had similar prediction ability of HU in both genders.

**Table 3:** AUC and cut-off values of four IR surrogates for prediction of HU.

Variables	AUC (95% CI)	Cut-off	Specificity	Sensitivity
Total				
TyG	0.66 (0.65-0.68)	8.44	0.62	0.62
TyG-BMI	0.67 (0.65-0.68)	224.16	0.54	0.70
TG/HDL-C	0.68 (0.67-0.69)	1.77	0.63	0.65
METS-IR	0.68 (0.66-0.69)	39.52	0.59	0.67
Males				
TyG	0.64 (0.62-0.66)	8.48	0.61	0.60
TyG-BMI	0.68 (0.67-0.70)	231.26	0.65	0.62
TG/HDL-C	0.64 (0.63-0.66)	1.78	0.55	0.67
METS-IR	0.68 (0.66-0.70)	39.52	0.61	0.66
Females				
TyG	0.66 (0.64-0.69)	8.44	0.64	0.61
TyG-BMI	0.71 (0.69-0.73)	241.00	0.62	0.70
TG/HDL-C	0.66 (0.63-0.68)	1.77	0.66	0.58
METS-IR	0.71 (0.68-0.73)	42.50	0.68	0.63

TyG, triglyceride glucose; TyG-BMI, triglyceride glucose with body mass index; TG/HDL-C, the ratio of triglycerides divided by high-density lipoprotein cholesterol; METS-IR, metabolic score for insulin resistance.

Then, we compared the prevalence of HU with escalating four IR surrogates. We found that the prevalence of HU tended to increase with the increase in the four IR surrogates (**Table S1–S3**). The cut-off value we obtained is a rather important turning point, above which the prevalence of HU almost doubles, both in the male and female population.

Similarly, the above results were validated for stability in the Bootstrap resampling (times = 500) analysis (**Figures S1–S3**).

## 4 Discussion

In this large-scale study that contains prospective and nationally-respresentative samples aged 18-80 years old of U.S. (N=7,743), it was found that the prevalence of HU was 32.18% for all (50.16% in males and 14.80% in females). There was an increased incidence of HU due to lifestyle and dietary changes, as well as aging (22). Therefore, early identification and control of IR in patients with HU before clinical symptoms may assist the management of HU and the prevention of its IR-driven comorbidities.

As an indirect method, measurement of IR surrogates was simple, economical, and convenient. Four surrogates, based on biochemical indexes of human body, were selected for IR, including TyG, TyG-BMI, TG/HDL-C and METS-IR. IR was closely related to glycolipid metabolism while previous researches have pointed out the significant association between these four surrogate indexes and the presence of IR (6, 12, 23–25). Our present study considered non-diabetic individuals in general population and expanded the sample size based on previous studies (7,743 *vs* 1,067) (26). This study not only further confirmed, in line with other existing studies, that IR surrogates were independently and positively correlated with the presence of HU, but also provided a simpler and more economical choice to distinguish IR status in non-diabetic patients with HU in clinic (12, 27).

Further ROC analysis proved that compared with TyG and TG/HDL-C, TyG-BMI and METS-IR excelled in IR discrimination in both gender groups. Given that obesity plays a vital role in the pathophysiology of IR (28, 29), combing obesity indicator with TyG should have better results theoretically. Our results are consistent with a previous research originated from NHANES, which indicated TyG-BMI had a significant and positive correlation with HU. METS-IR is a novel index that combines non-insulin fasting laboratory values and anthropometric measurements, both of which can be easily obtained in primary care evaluation, to assess insulin sensitivity and detect IR cases (8, 30). However, studies of Liu et al. had different results, suggesting that TG/HDL-C was most strongly associated with HU (11). Such a discrepancy may be attributed to: firstly, the level of insulin secretion and sensitivity greatly differs by ethnicity (31); secondly, obesity, which plays an important role in IR; thirdly, difference in sample size. All in all, ethnic-based, larger-scale studies are needed to elucidate this disparity.

In addition, we also found gender differences in these four indicators of IR and HU: they showed better predictive effect in females than males. Similar findings were reported by a study conducted in 2020 which revealed that elevated UA was associated with a higher risk of IR and such an association was more pronounced in female patients. This difference may be attributable to different sex hormones and adipokines, which cause more insulin-sensitive characteristics of females (32). Considering previous research results, it is agreed that more attention should be paid to the application of IR substitutes as predictors of HU in females.

This study has both advantages and limitations. Present study is the first large-scale research with nationally-representative samples to examine the association between these four non-insulin-based indicators of IR and HU, which increased the statistical strength and confirmed the reliability of reported results. However, several limitations should also be noted. First of all, the causal relationship between these IR indicators and HU cannot be well explained by this study. Secondly, retrospective data in our study may have recall bias. Thirdly, the study population was solely from the United States, for which conclusions may not be generalizable.

## 5 Conclusion

In this study, it was found that the risk of HU was positively associated with the elevation of TyG, TyG-BMI, TG/HDL-C and METS-IR in a large-scale population of U.S. Among the four IR surrogates, TyG-BMI and METS-IR had pronounced discrimination ability to HU. Moreover, all four IR surrogates had better prediction ability for HU in females. To sum up, four IR surrogates are recommended as complementary markers for the assessment of HU risk both in clinic and in future epidemiological studies in non-diabetic populations. Yet more researches are in need to provide reference for different gender groups.

## Data availability statement

The datasets presented in this study can be found in online repositories. The names of the repository/repositories and accession number(s) can be found below: https://www.cdc.gov/nchs/nhanes/index.htm.

## Ethics statement

The studies involving human participants were reviewed and approved by National Centre for Health Statistics Institutional Ethics Review Board. The patients/participants provided their written informed consent to participate in this study.

## Author contributions

HW: writing-most of manuscript, data curation and processing. JZ: writing-part of the manuscript, data curation. YP: writing-part of the manuscript, data processing. SQ, HL and YT: software, writing—review and editing, and supervision. ZT: methodology, writing—review and editing, and supervision. All authors contributed to the article and approved the submitted version.
