# Supplementary material for: Comparison of different insulin resistance surrogates to predict hyperuricemia among U.S. non-diabetic adults
Source: Front Endocrinol (Lausanne). 2022 Dec 15;13:1028167. doi: 10.3389/fendo.2022.1028167 (PMC9797589; doi:10.3389/fendo.2022.1028167)
Supplement: Supplementary file 1 [file DataSheet_1.docx]

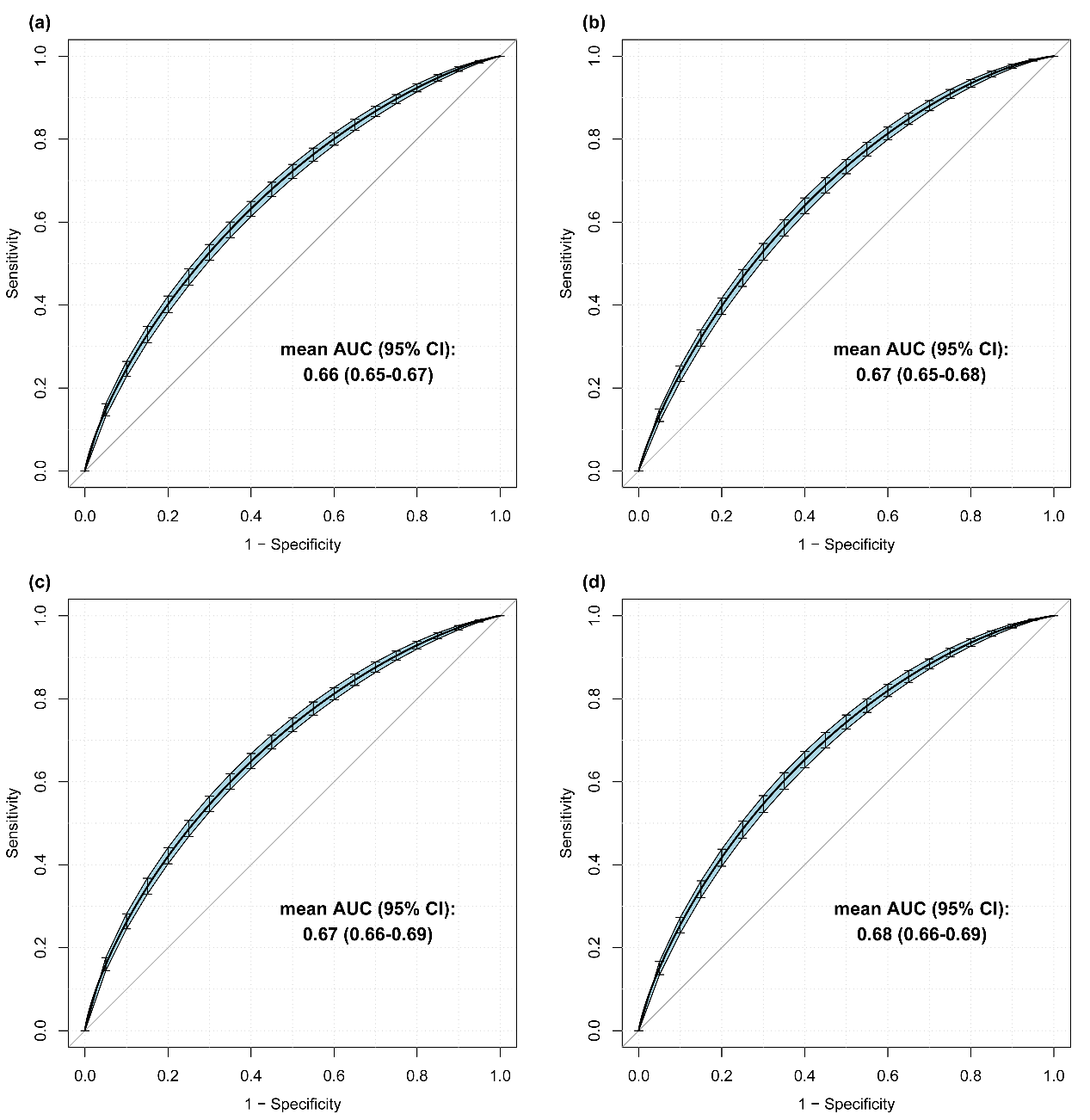


**Figure S1.** ROC by using Bootstrap resampling (times = 500) for different IR surrogates ((a)TyG; (b)TyG-BMI; (c)TG/HDL-C; (d)METS-IR) to predict HU. Shading shows the bootstrap estimated 95% CI with the AUC.

**Abbreviations:** ROC, receiver operating characteristic; AUC, area under the curve; CI, confidence interval; HU, hyperuricemia; TyG, triglyceride glucose; TyG-BMI, triglyceride glucose with body mass index; TG/HDL-C, the ratio of triglycerides divided by high-density lipoprotein cholesterol; METS-IR, metabolic score for insulin resistance.


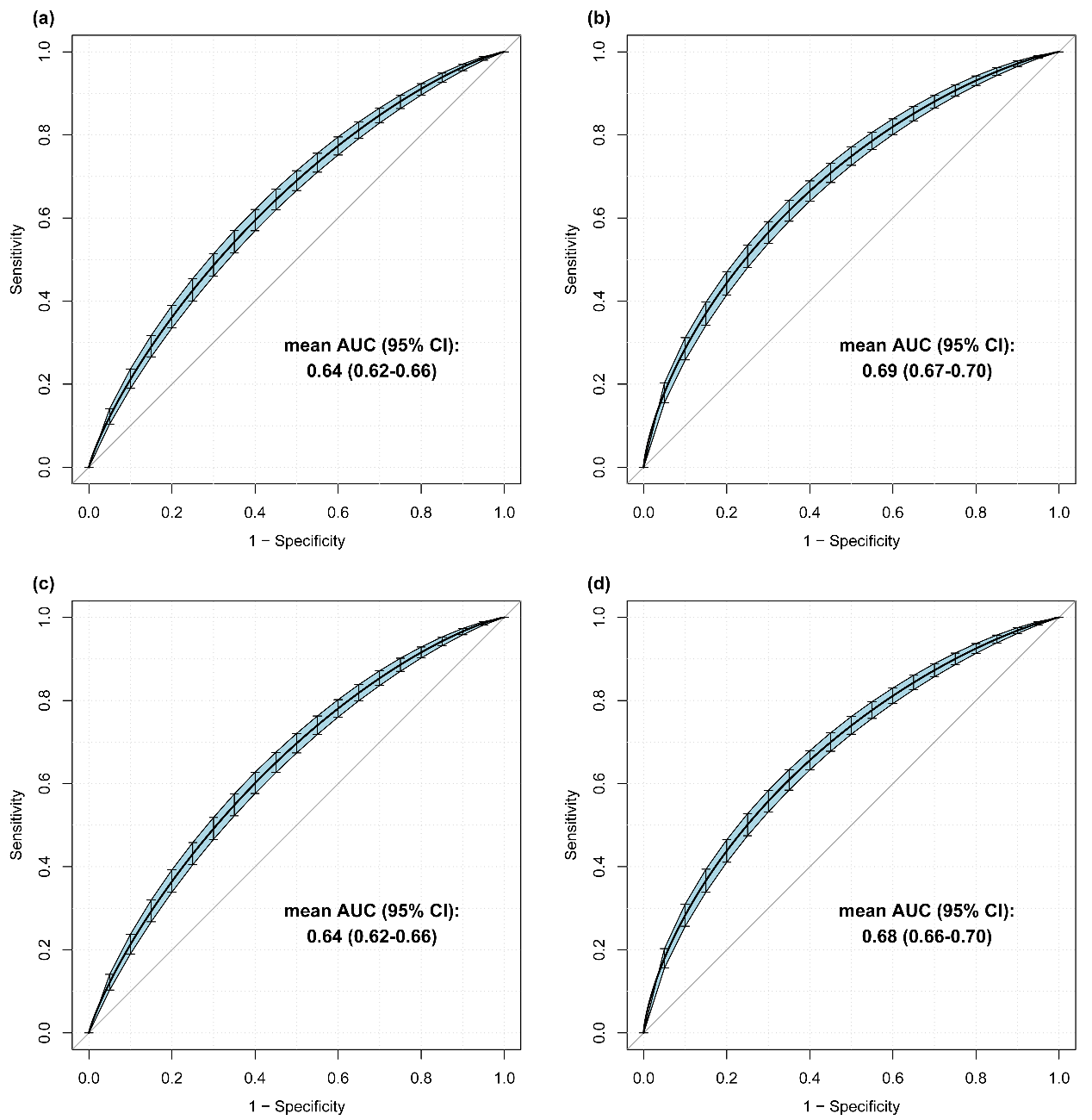


**Figure S2.** ROC by using Bootstrap resampling (times = 500) in males for different IR surrogates ((a)TyG; (b)TyG-BMI; (c)TG/HDL-C; (d)METS-IR) to predict HU. Shading shows the bootstrap estimated 95% CI with the AUC.

**Abbreviations:** ROC, receiver operating characteristic; AUC, area under the curve; CI, confidence interval; HU, hyperuricemia; TyG, triglyceride glucose; TyG-BMI, triglyceride glucose with body mass index; TG/HDL-C, the ratio of triglycerides divided by high-density lipoprotein cholesterol; METS-IR, metabolic score for insulin resistance.


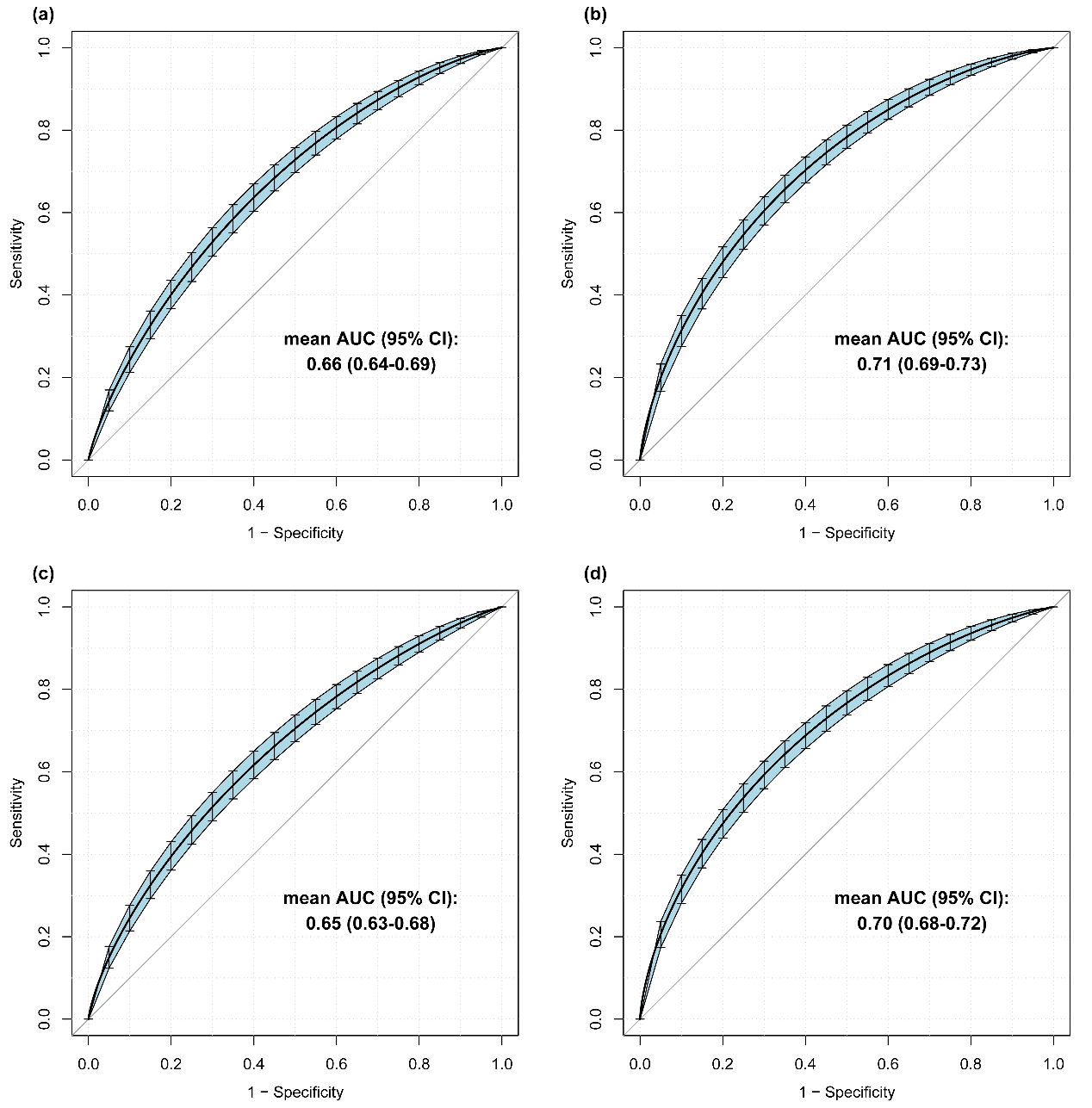


**Figure S3.** ROC by using Bootstrap resampling (times = 500) in females for different IR surrogates ((a)TyG; (b)TyG-BMI; (c)TG/HDL-C; (d)METS-IR) to predict HU. Shading shows the bootstrap estimated 95% CI with the AUC.

**Abbreviations:** ROC, receiver operating characteristic; AUC, area under the curve; CI, confidence interval; HU, hyperuricemia; TyG, triglyceride glucose; TyG-BMI, triglyceride glucose with body mass index; TG/HDL-C, the ratio of triglycerides divided by high-density lipoprotein cholesterol; METS-IR, metabolic score for insulin resistance.
