# Supplementary material for: Comparison of different insulin resistance surrogates to predict hyperuricemia among U.S. non-diabetic adults
Source: Front Endocrinol (Lausanne). 2022 Dec 15;13:1028167. doi: 10.3389/fendo.2022.1028167 (PMC9797589; doi:10.3389/fendo.2022.1028167)
Supplement: Supplementary file 2 [file DataSheet_2.docx]

**Table S1. The tendency of the prevalence of HU as the escalation of four IR surrogates**

| **Variables** | **Without HU** | **With HU** | **The prevalence of HU (%)** | **P-value** |
| --- | --- | --- | --- | --- |
| **TyG** |  |  |  | <0.01 |
| ＜8.44 | 3232 | 943 | 37.84 |  |
| ≥8.44 | 2019 | 1549 | 62.16 |  |
| **TyG-BMI** |  |  |  | <0.01 |
| ＜224.16 | 2833 | 741 | 29.74 |  |
| ≥224.16 | 2418 | 1751 | 70.26 |  |
| **TG/HDL-C** |  |  |  | <0.01 |
| ＜1.77 | 3272 | 878 | 35.23 |  |
| ≥1.77 | 1979 | 1614 | 64.77 |  |
| **METS-IR** |  |  |  | <0.01 |
| ＜39.52 | 3119 | 823 | 33.03 |  |
| ≥39.52 | 2132 | 1669 | 66.97 |  |

**Abbreviations:** HU, hyperuricemia; IR, insulin resistance; TyG, triglyceride glucose; TyG-BMI, triglyceride glucose with body mass index; TG/HDL-C, the ratio of triglycerides divided by high-density lipoprotein cholesterol; METS-IR, metabolic score for insulin resistance.

**Table S2. The tendency of the prevalence of HU as the escalation of four IR surrogates** **in males**

| **Variables** | **Without HU** | **With HU** | **The prevalence of HU (%)** | **P-value** |
| --- | --- | --- | --- | --- |
| **TyG** |  |  |  | <0.01 |
| ＜8.48 | 1133 | 760 | 40.15 |  |
| ≥8.48 | 720 | 1133 | 59.85 |  |
| **TyG-BMI** |  |  |  | <0.01 |
| ＜231.26 | 1204 | 718 | 37.93 |  |
| ≥231.26 | 649 | 1175 | 62.07 |  |
| **TG/HDL-C** |  |  |  | <0.01 |
| ＜1.78 | 1028 | 630 | 33.28 |  |
| ≥1.78 | 825 | 1263 | 66.72 |  |
| **METS-IR** |  |  |  | <0.01 |
| ＜39.52 | 1122 | 646 | 34.13 |  |
| ≥39.52 | 731 | 1247 | 65.87 |  |

**Abbreviations:** HU, hyperuricemia; IR, insulin resistance; TyG, triglyceride glucose; TyG-BMI, triglyceride glucose with body mass index; TG/HDL-C, the ratio of triglycerides divided by high-density lipoprotein cholesterol; METS-IR, metabolic score for insulin resistance.

**Table S3. The tendency of the prevalence of HU as the escalation of four IR surrogates** **in females**

| **Variables** | **Without HU** | **With HU** | **The prevalence of HU (%)** | **P-value** |
| --- | --- | --- | --- | --- |
| **TyG** |  |  |  | <0.01 |
| ＜8.44 | 2163 | 236 | 39.40 |  |
| ≥8.44 | 1235 | 363 | 60.60 |  |
| **TyG-BMI** |  |  |  | <0.01 |
| ＜241.00 | 2119 | 182 | 30.38 |  |
| ≥241.00 | 1279 | 417 | 69.62 |  |
| **TG/HDL-C** |  |  |  | <0.01 |
| ＜1.77 | 2251 | 254 | 42.40 |  |
| ≥1.77 | 1147 | 345 | 57.60 |  |
| **METS-IR** |  |  |  | <0.01 |
| ＜42.50 | 2296 | 221 | 36.89 |  |
| ≥42.50 | 1102 | 378 | 63.11 |  |

**Abbreviations:** HU, hyperuricemia; IR, insulin resistance; TyG, triglyceride glucose; TyG-BMI, triglyceride glucose with body mass index; TG/HDL-C, the ratio of triglycerides divided by high-density lipoprotein cholesterol; METS-IR, metabolic score for insulin resistance.

**Table S4. Mean AUC and cut-off values of four IR surrogates for prediction of HU (after 500 times Bootstrap resampling)**

| **Variables** | **Mean AUC (95% CI)** | **Cut-off** | **Specificity** | **Sensitivity** |
| --- | --- | --- | --- | --- |
| **Total** | | | | |
| **TyG** | 0.66 (0.65-0.67) | 8.44 | 0.62 | 0.62 |
| **TyG-BMI** | 0.67 (0.65-0.68) | 224.16 | 0.54 | 0.70 |
| **TG/HDL-C** | 0.67 (0.66-0.69) | 1.77 | 0.63 | 0.65 |
| **METS-IR** | 0.68 (0.66-0.69) | 39.52 | 0.59 | 0.67 |
| **Males** | | | | |
| **TyG** | 0.64 (0.62-0.66) | 8.48 | 0.61 | 0.60 |
| **TyG-BMI** | 0.69 (0.67-0.70) | 231.26 | 0.65 | 0.62 |
| **TG/HDL-C** | 0.64 (0.62-0.66) | 1.78 | 0.55 | 0.67 |
| **METS-IR** | 0.68 (0.66-0.70) | 39.52 | 0.61 | 0.66 |
| **Females** | | | | |
| **TyG** | 0.66 (0.64-0.69) | 8.44 | 0.64 | 0.61 |
| **TyG-BMI** | 0.71 (0.69-0.73) | 241.00 | 0.62 | 0.70 |
| **TG/HDL-C** | 0.65 (0.63-0.68) | 1.77 | 0.66 | 0.58 |
| **METS-IR** | 0.70 (0.68-0.72) | 42.50 | 0.68 | 0.63 |

**Abbreviations:** TyG, triglyceride glucose; TyG-BMI, triglyceride glucose with body mass index; TG/HDL-C, the ratio of triglycerides divided by high-density lipoprotein cholesterol; METS-IR, metabolic score for insulin resistance.

**Methods**

**S1 Programming language of Bootstrap resampling**

**S1.1 ROC by using Bootstrap resampling for different IR surrogates to predict HU**

R.Version4RUN<-343;

R.LibLocation <- "C:/Users/Administrator/AppData/Roaming/EmpowerRCH/R343/library"

Sys.setlocale("LC_TIME", "C")

library(doBy,lib.loc=R.LibLocation)

library(plotrix,lib.loc=R.LibLocation)

library(stringi,lib.loc=R.LibLocation)

library(stringr,lib.loc=R.LibLocation)

library(survival,lib.loc=R.LibLocation)

library(rms,lib.loc=R.LibLocation)

library(nnet,lib.loc=R.LibLocation)

library(car,lib.loc=R.LibLocation)

library(mgcv,lib.loc=R.LibLocation)

pdfwd<-6; pdfht<-6

setwd("F:/data_process/IR.UA2/PROJ29_24_tbl")

load("F:/data_process/IR.UA2/wt.Rdata")

if (length(which(ls()=="EmpowerStatsR"))==0) EmpowerStatsR<-get(ls()[1])

names(EmpowerStatsR)<-toupper(names(EmpowerStatsR))

rankvar <- function(var, num) {

qprobs <- 1/num

if (num>2) {for (i in (2:(num-1))) {qprobs <- c(qprobs, 1/num * i) }}

outvar <- rep(0, times=length(var))

outvar[is.na(var)] <- NA

cutpoints <- quantile(var,probs=qprobs, na.rm=TRUE)

for (k in (1:length(cutpoints))) {outvar[var>=cutpoints[k]] <- k}

tmp<-c(min(var,na.rm=TRUE),cutpoints,max(var,na.rm=TRUE))

names(tmp)<-c("Min",names(cutpoints),"Max")

print(tmp)

return(outvar)

}

recodevar <- function (var,oldcode,newcode) {

tmp.v <- var

nc.tmp <- length(oldcode)

for (i in (1:nc.tmp)) {tmp.v[(var==oldcode[i])]=newcode[i]}

if (is.factor(tmp.v)) {tmp.v1<-as.numeric(as.character(tmp.v))} else {tmp.v1<-as.numeric(tmp.v)}

rm(tmp.v); return(tmp.v1)

}

attach(EmpowerStatsR)

sink("F:/data_process/IR.UA2/datastep/PROJ29_datastep.lst")

print("Create new variable: POPSLT.CRITERIA1")

POPSLT.CRITERIA1<-ifelse((!is.na(X8) & !is.na(X14)),1,0)

EmpowerStatsR<-cbind(EmpowerStatsR,POPSLT.CRITERIA1)

POPSLT.STEP1<-POPSLT.CRITERIA1

print("Create new variable: POPSLT.CRITERIA2")

POPSLT.CRITERIA2<-ifelse((!is.na(X8) & !is.na(X14) & (!is.na(X20) & (X20!=2))),1,0)

EmpowerStatsR<-cbind(EmpowerStatsR,POPSLT.CRITERIA2)

print("Creating new variable: POPSLT.STEP2")

POPSLT.STEP2<-ifelse((POPSLT.STEP1==1 & POPSLT.CRITERIA2==1),1,0)

EmpowerStatsR<-cbind(EmpowerStatsR,POPSLT.STEP2)

print("Create new variable: POPSLT.CRITERIA3")

POPSLT.CRITERIA3<-ifelse((!is.na(X8) & !is.na(X14) & (!is.na(X20) & (X20!=2)) & (X3<10.5)),1,0)

EmpowerStatsR<-cbind(EmpowerStatsR,POPSLT.CRITERIA3)

print("Creating new variable: POPSLT.STEP3")

POPSLT.STEP3<-ifelse((POPSLT.STEP2==1 & POPSLT.CRITERIA3==1),1,0)

EmpowerStatsR<-cbind(EmpowerStatsR,POPSLT.STEP3)

print("Creating new variable: HYPER.UA1")

HYPER.UA1<-rep(NA,times=nrow(EmpowerStatsR))

tmp<- (is.na(HYPER.UA1) & (X5==1 & X17>=7))

tmp[is.na(tmp)]<-FALSE

HYPER.UA1[tmp]<-1

tmp<- (is.na(HYPER.UA1) & (X5==2 & X17>=6))

tmp[is.na(tmp)]<-FALSE

HYPER.UA1[tmp]<-1

tmp<-(is.na(HYPER.UA1))

HYPER.UA1[tmp]<-0

EmpowerStatsR<-cbind(EmpowerStatsR,HYPER.UA1)

print("Creating new variable: TYG.BMI")

TYG.BMI<- X3*X8

EmpowerStatsR<-cbind(EmpowerStatsR,TYG.BMI)

print("Creating new variable: TG.HDL")

TG.HDL <- X2/X14

EmpowerStatsR<-cbind(EmpowerStatsR,TG.HDL)

print("Creating new variable: MET1")

MET1 <- log(2*X15+X2)*X8

EmpowerStatsR<-cbind(EmpowerStatsR,MET1)

print("Creating new variable: MET2")

MET2 <- log(X14)

EmpowerStatsR<-cbind(EmpowerStatsR,MET2)

print("Creating new variable: X3.Q4")

X3.Q4<- rankvar(X3,4)

EmpowerStatsR<-cbind(EmpowerStatsR,X3.Q4)

print("Creating new variable: TYG.BMI.Q4")

TYG.BMI.Q4<- rankvar(TYG.BMI,4)

EmpowerStatsR<-cbind(EmpowerStatsR,TYG.BMI.Q4)

print("Creating new variable: TG.HDL.Q4")

TG.HDL.Q4<- rankvar(TG.HDL,4)

EmpowerStatsR<-cbind(EmpowerStatsR,TG.HDL.Q4)

print("Creating new variable: MET.IR")

MET.IR <- MET1/MET2

EmpowerStatsR<-cbind(EmpowerStatsR,MET.IR)

print("Creating new variable: MET.IR.Q4")

MET.IR.Q4<- rankvar(MET.IR,4)

EmpowerStatsR<-cbind(EmpowerStatsR,MET.IR.Q4)

print("Creating new variable: X17.CS")

X17.CS<- 0+(X17<6)

X17.CS[is.na(X17)]<-NA

EmpowerStatsR<-cbind(EmpowerStatsR,X17.CS)

print("Creating new variable: X17.CS.NEW")

X17.CS.NEW<- recodevar(X17.CS,c(0,1),c(1,0))

EmpowerStatsR<-cbind(EmpowerStatsR,X17.CS.NEW)

print("Creating new variable: X3.CS")

X3.CS<- 0+(X3>=8.44)

X3.CS[is.na(X3)]<-NA

EmpowerStatsR<-cbind(EmpowerStatsR,X3.CS)

print("Creating new variable: TYG.BMI.CS")

TYG.BMI.CS<- 0+(TYG.BMI>=224.16)

TYG.BMI.CS[is.na(TYG.BMI)]<-NA

EmpowerStatsR<-cbind(EmpowerStatsR,TYG.BMI.CS)

print("Creating new variable: TG.HDL.CS")

TG.HDL.CS<- 0+(TG.HDL>=1.77)

TG.HDL.CS[is.na(TG.HDL)]<-NA

EmpowerStatsR<-cbind(EmpowerStatsR,TG.HDL.CS)

print("Creating new variable: MET.IR.CS")

MET.IR.CS<- 0+(MET.IR>=39.52)

MET.IR.CS[is.na(MET.IR)]<-NA

EmpowerStatsR<-cbind(EmpowerStatsR,MET.IR.CS)

print("Creating new variable: X3.CS1")

X3.CS1<- 0+(X3>=8.48)

X3.CS1[is.na(X3)]<-NA

EmpowerStatsR<-cbind(EmpowerStatsR,X3.CS1)

print("Creating new variable: TYG.BMI.CS1")

TYG.BMI.CS1<- 0+(TYG.BMI>=231.26)

TYG.BMI.CS1[is.na(TYG.BMI)]<-NA

EmpowerStatsR<-cbind(EmpowerStatsR,TYG.BMI.CS1)

print("Creating new variable: TG.HDL.CS1")

TG.HDL.CS1<- 0+(TG.HDL>=1.78)

TG.HDL.CS1[is.na(TG.HDL)]<-NA

EmpowerStatsR<-cbind(EmpowerStatsR,TG.HDL.CS1)

print("Creating new variable: MET.IR.CS1")

MET.IR.CS1<- 0+(MET.IR>=39.52)

MET.IR.CS1[is.na(MET.IR)]<-NA

EmpowerStatsR<-cbind(EmpowerStatsR,MET.IR.CS1)

print("Creating new variable: X3.CS2")

X3.CS2<- 0+(X3>=8.44)

X3.CS2[is.na(X3)]<-NA

EmpowerStatsR<-cbind(EmpowerStatsR,X3.CS2)

print("Creating new variable: TYG.BMI.CS2")

TYG.BMI.CS2<- 0+(TYG.BMI>=241)

TYG.BMI.CS2[is.na(TYG.BMI)]<-NA

EmpowerStatsR<-cbind(EmpowerStatsR,TYG.BMI.CS2)

print("Creating new variable: TG.HDL.CS2")

TG.HDL.CS2<- 0+(TG.HDL>=1.77)

TG.HDL.CS2[is.na(TG.HDL)]<-NA

EmpowerStatsR<-cbind(EmpowerStatsR,TG.HDL.CS2)

print("Creating new variable: MET.IR.CS2")

MET.IR.CS2<- 0+(MET.IR>=42.5)

MET.IR.CS2[is.na(MET.IR)]<-NA

EmpowerStatsR<-cbind(EmpowerStatsR,MET.IR.CS2)

rm(HYPER.UA1,TYG.BMI,TG.HDL,MET1,MET2,X3.Q4,TYG.BMI.Q4,TG.HDL.Q4,MET.IR,MET.IR.Q4,X17.CS,X17.CS.NEW,X3.CS,TYG.BMI.CS,TG.HDL.CS,MET.IR.CS,X3.CS1,TYG.BMI.CS1,TG.HDL.CS1,MET.IR.CS1,X3.CS2,TYG.BMI.CS2,TG.HDL.CS2,MET.IR.CS2)

rm(POPSLT.CRITERIA1,POPSLT.STEP1,POPSLT.CRITERIA2,POPSLT.STEP2,POPSLT.CRITERIA3,POPSLT.STEP3)

detach(EmpowerStatsR)

sink()

vname<-c("_N_","_STAT_","_TOTAL_","X1","X2","X3","X4")

vlabel<-c("sample size (%)","statistic","total","seqn","TG","TyG","age")

vname<-c(vname,"X5","X6","X7","X8","X9","X10")

vlabel<-c(vlabel,"sex","eth","nhs_wt","BMI","smoke","drink")

vname<-c(vname,"X11","X12","X13","X14","X15")

vlabel<-c(vlabel,"PA_total_MET","TC","LDL-C","HDL-C","FPG")

vname<-c(vname,"X16","X17","X18","X19","X20","X20.1","X20.2","X20.3")

vlabel<-c(vlabel,"eGFR","UA","sbp","dbp","DM"," no"," yes"," pre")

vname<-c(vname,"X21","X21.1","X21.2","X22","X22.1","X22.2")

vlabel<-c(vlabel,"Hyperlipidemia"," no"," yes","Hypertension"," no"," yes")

vname<-c(vname,"X23","X23.1","X23.2","X24","X24.1","X24.2")

vlabel<-c(vlabel,"Diabetic"," no"," yes","Hyperlipidemic"," no"," yes")

vname<-c(vname,"X25","X25.1","X25.2","X26","X26.1","X26.2","X26.3")

vlabel<-c(vlabel,"Hypertensive"," no"," yes","sdmvpsu"," 1"," 2"," 3")

vname<-c(vname,"X27","X28","X29","X29.1","X29.2","X29.3")

vlabel<-c(vlabel,"sdmvstra","wtmec2yr","education"," less than high school"," high school"," more than high school")

vname<-c(vname,"HYPER.UA1","HYPER.UA1.0","HYPER.UA1.1","TYG.BMI")

vlabel<-c(vlabel,"HYPER.UA1"," 0"," 1","TyG * BMI")

vname<-c(vname,"TG.HDL","MET1","MET2","X3.Q4","X3.Q4.0","X3.Q4.1","X3.Q4.2","X3.Q4.3")

vlabel<-c(vlabel,"TG.HDL","MET1","MET2","TyG Quartered"," Q1"," Q2"," Q3"," Q4")

vname<-c(vname,"TYG.BMI.Q4","TYG.BMI.Q4.0","TYG.BMI.Q4.1","TYG.BMI.Q4.2","TYG.BMI.Q4.3")

vlabel<-c(vlabel,"TyG * BMI Quartered"," Q1"," Q2"," Q3"," Q4")

vname<-c(vname,"TG.HDL.Q4","TG.HDL.Q4.0","TG.HDL.Q4.1","TG.HDL.Q4.2","TG.HDL.Q4.3")

vlabel<-c(vlabel,"TG.HDL Quartered"," Q1"," Q2"," Q3"," Q4")

vname<-c(vname,"MET.IR","MET.IR.Q4","MET.IR.Q4.0","MET.IR.Q4.1","MET.IR.Q4.2","MET.IR.Q4.3")

vlabel<-c(vlabel,"MET.IR","MET.IR Quartered"," Q1"," Q2"," Q3"," Q4")

vname<-c(vname,"X17.CS","X17.CS.0","X17.CS.1","X17.CS.NEW","X17.CS.NEW.0","X17.CS.NEW.1")

vlabel<-c(vlabel,"UA group"," >=6"," <6","UA"," 0"," 1")

vname<-c(vname,"X3.CS","X3.CS.0","X3.CS.1","TYG.BMI.CS","TYG.BMI.CS.0","TYG.BMI.CS.1")

vlabel<-c(vlabel,"TyG group"," <8.44"," >=8.44","TyG * BMI group"," <224.16"," >=224.16")

vname<-c(vname,"TG.HDL.CS","TG.HDL.CS.0","TG.HDL.CS.1","MET.IR.CS","MET.IR.CS.0","MET.IR.CS.1")

vlabel<-c(vlabel,"TG.HDL group"," <1.77"," >=1.77","MET.IR group"," <39.52"," >=39.52")

vname<-c(vname,"X3.CS1","X3.CS1.0","X3.CS1.1","TYG.BMI.CS1","TYG.BMI.CS1.0","TYG.BMI.CS1.1")

vlabel<-c(vlabel,"TyG group"," <8.48"," >=8.48","TyG * BMI group"," <231.26"," >=231.26")

vname<-c(vname,"TG.HDL.CS1","TG.HDL.CS1.0","TG.HDL.CS1.1")

vlabel<-c(vlabel,"TG.HDL group"," <1.78"," >=1.78")

vname<-c(vname,"MET.IR.CS1","MET.IR.CS1.0","MET.IR.CS1.1")

vlabel<-c(vlabel,"MET.IR group"," <39.52"," >=39.52")

vname<-c(vname,"X3.CS2","X3.CS2.0","X3.CS2.1","TYG.BMI.CS2","TYG.BMI.CS2.0","TYG.BMI.CS2.1")

vlabel<-c(vlabel,"TyG group"," <8.44"," >=8.44","TyG * BMI group"," <241"," >=241")

vname<-c(vname,"TG.HDL.CS2","TG.HDL.CS2.0","TG.HDL.CS2.1")

vlabel<-c(vlabel,"TG.HDL group"," <1.77"," >=1.77")

vname<-c(vname,"MET.IR.CS2","MET.IR.CS2.0","MET.IR.CS2.1")

vlabel<-c(vlabel,"MET.IR group"," <42.5"," >=42.5")

slt.vname<-c()

library(pROC,lib.loc=R.LibLocation)

library(survivalROC,lib.loc=R.LibLocation)

library(plyr,lib.loc=R.LibLocation)

library(rms,lib.loc=R.LibLocation)

library(rmda,lib.loc=R.LibLocation)

library(Hmisc,lib.loc=R.LibLocation)

ofname<-"PROJ29_24_tbl";

attach(EmpowerStatsR);WD<-EmpowerStatsR[(POPSLT.CRITERIA2==1),];detach(EmpowerStatsR);

wd.subset<-paste("Use subset of data:","POPSLT.CRITERIA2==1");svy.DSN.YN <- FALSE;

weights<-1;weights.var <- NA;

WD<-cbind(WD,weights); WD<-WD[!is.na(weights),];

title<-"Diagnostic test with ROC analysis - resampling";

attach(WD)

subjvname<-NA;

xv<-cbind(X3,TYG.BMI,TG.HDL,MET.IR);

xvname<-c('X3','TYG.BMI','TG.HDL','MET.IR');

xvar<-c('X3','TYG_BMI','TG_HDL','MET_IR');

xlv<-c(0,0,0,0);

sxf<-NA;

svname<-NA; sv<-NA; slv<-NA;

av<-NA; avname<-NA; avlbl<-NA; nadj<-0; alv<-NA;

timev<-NA; timevname<-NA;

bv<-NA; bvar<-NA;

colv<-X17.CS.NEW;colvname<-"X17.CS.NEW";

v.start<-NA; vname.start<-NA;

v.stop<-NA; vname.stop<-NA;

par1<-1;dec<-4;parm<-c(1,NA, NA, NA, 0);

if (!exists("pdfwd")) pdfwd<-6;

if (!exists("pdfht")) pdfht<-6;

##R package## pROC survivalROC plyr rms rmda Hmisc ##R package##;

pvformat<-function(p,dec) {

pp <- sprintf(paste("%.",dec,"f",sep=""),as.numeric(p))

if (is.matrix(p)) {pp<-matrix(pp, nrow=nrow(p)); colnames(pp)<-colnames(p);rownames(pp)<-rownames(p);}

lw <- paste("<",substr("0.00000000000",1,dec+1),"1",sep="");

pp[as.numeric(p)<(1/10^dec)]<-lw

return(pp)

}

numfmt<-function(p,dec) {

if (is.list(p)) p<-as.matrix(p)

pp <- sprintf(paste("%.",dec,"f",sep=""),as.numeric(p))

if (is.matrix(p)) {pp<-matrix(pp, nrow=nrow(p));colnames(pp)<-colnames(p);rownames(pp)<-rownames(p);}

pp[as.numeric(p)>10000000]<- "inf."

pp[is.na(p) | gsub(" ","",p)==""]<- ""

pp[p=="-Inf"]<-"-Inf"

pp[p=="Inf"]<-"Inf"

return(pp)

}

rocplot<-function(x,xb) {

xlm<-c(0,1);

x1 <- 1-x$specificities; y1<-x$sensitivities;

if (x$percent) {x1<-x1/100; y1<-y1/100;}

plot(x1, y1, xlim=c(0, 1), ylim=c(0, 1), xlab="1 - Specificity", ylab="Sensitivity",

asp=0.9, mar=c(4, 4, 2, 2)+.1, mgp=c(2.5, 1, 0), col=par("col"),

lty=par("lty"), lwd=2, type="l", main=paste(xb, "\nAUC =", round(x$auc,3)));

abline(a=0,b=1,col="darkgrey",lty=1,lwd=1);

}

rocplot2<-function(x,xb,mainb) {

n=length(x); xlm<-c(0,1); ccol<-(1:n)

x1 <- 1-x[[1]]$specificities; y1<-x[[1]]$sensitivities; auc<-x[[1]]$auc

if (x[[1]]$percent) {x1<-x1/100; y1<-y1/100;}

if (n==1) {mb<-paste(xb[1],"\nAUC =", round(auc,3));

} else {mb<-mainb;}

plot(x1, y1, xlim=xlm, ylim=xlm,

xlab="1 - Specificity", ylab="Sensitivity",

asp=0.9, mar=c(4, 4, 2, 2)+.1, mgp=c(2.5, 1, 0),

col=1, lty=1, lwd=2, type="l", main=mb);

abline(a=0,b=1,col="darkgrey",lty=1,lwd=1);

for (k in (2:n)) {

auc<-c(auc,x[[k]]$auc)

par(new=T)

xk <- 1-x[[k]]$specificities; yk<-x[[k]]$sensitivities

if (x[[k]]$percent) {xk<-xk/100; yk<-yk/100;}

plot(xk, yk, xlim=xlm, ylim=xlm, xlab="", ylab="",

asp=0.9, mar=c(4, 4, 2, 2)+.1, mgp=c(2.5, 1, 0),

col=k, lty=1, lwd=2, type="l", main="");

}

if (n>1) {

mlen<-min(max(nchar(xb)),15);

mspace<-paste(rep(" ",mlen),collapse="")

xb1<-substr(paste(xb,mspace),1,mlen)

tauc<-paste(xb1,pvformat(auc,3),sep=": ")

ord<-order(auc,decreasing=TRUE)

tauc<-tauc[ord]; clr<-(1:n)[ord]

legend(0.6,0.4,tauc,title="AUC",col=clr,lty=1,lwd=1,bty="n")

}

}

diagss<-function(d,x,xb,xlb,ylb,pngf) {

cmp<- !is.na(d) & !is.na(x); d<-d[cmp]; x<-x[cmp]

d0<-d; d<-2-d; ndL <- length(levels(factor(d))); nxL <- length(levels(factor(x)));

if (ndL!=2 | nxL<2) return (c(xb,rep(" ",6)))

lr <- rep(NA,nxL)

if (nxL==2) {x<-2-x; xlb<-c(xlb[2],xlb[1])}

t0<-table(x,d); ctot<-apply(t0,2,sum); rtot<-apply(t0,1,sum)

for (i in (1:nrow(t0))) lr[i] <- (t0[i,1]/ctot[1]) / (t0[i,2]/ctot[2])

tt <- rbind(c(xb, " "," "," "),cbind(xlb,t0,format(round(lr,dec),nsmall=dec)))

if (nxL==2) {

sen <- t0[1,1]/ctot[1]; spe<- t0[2,2]/ctot[2]

ppv <- t0[1,1]/rtot[1]; npv<- t0[2,2]/rtot[2]

acc<-(t0[1,1]+t0[2,2])/(ctot[1]+ctot[2])

plr<-sen/(1-spe); nlr<-(1-sen)/spe; nnd<-1/(sen+spe-1); dor<-plr/nlr

tt<-cbind(tt,rbind(format(round(c(sen,spe,ppv,npv,acc,plr,nlr,dor,nnd),dec),nsmall=dec),matrix(" ",nrow=nxL,ncol=9)))

} else {

tmp.glm<-glm(d0~factor(x),family=binomial(link="logit"))

roc0<-roc(d0,predict(tmp.glm))

a.tss<-roc2xls(roc0,d0)

colnames(a.tss)<-c("threshold","specificity","sensitivity","accuracy","positive-LLR","negative-LLR","diagnose-OR","N-for-diagnose","postive-pv","negative-pv","a","b","c","d")

bb<-format(round(a.tss[which.max(a.tss[,2]+a.tss[,3]),c(3,2,9,10,4,5,6,7,8)],dec),nsmall=dec)

xlsfname<-paste(pngf,"_roc.xls",sep="")

write.table(a.tss,file=xlsfname,row.names=FALSE,col.names=TRUE,sep="\t",append=FALSE,quote=FALSE)

png(paste(ofname,"_raw.png",sep="")); rocplot(roc0,xb); dev.off()

pdf(paste(ofname,"_raw.pdf",sep=""), width=pdfwd, height=pdfht, family="Helvetica"); rocplot(roc0,xb); dev.off()

tt<-cbind(tt,rbind(bb,matrix(" ",nrow=(nrow(tt)-1),ncol=9)))

}

return(tt)

}

roc2xls<-function(roc0,d) {

a.tss<-t(coords(roc0,"all",ret=c("threshold", "specificity", "sensitivity"), transpose=TRUE))

bdac<-table(d); t.bd<-bdac[1]; t.ac<-bdac[2]

t.a<-a.tss[,3]*t.ac; t.c<-t.ac-t.a; t.d<-a.tss[,2]*t.bd; t.b<-t.bd-t.d;

acc<-(t.a+t.d)/(t.ac+t.bd)

plr<-(t.a/(t.a+t.c))/(t.b/(t.b+t.d))

nlr<-(t.c/(t.a+t.c))/(t.d/(t.b+t.d))

dor<-plr/nlr

nnd<-1/(t.a/(t.a+t.c)-t.b/(t.b+t.d))

ppv<-t.a/(t.a+t.b)

npv<-t.d/(t.c+t.d)

a.tss<-cbind(a.tss,acc,plr,nlr,dor,nnd,ppv,npv,t.a,t.b,t.c,t.d)

return(a.tss)

}

glm2formula<-function(mdl) {

coe<-summary(mdl)$coefficients;

tmp<-rownames(coe);

tmp<-gsub(")","=",tmp)

tmp[substr(tmp,1,7)=="factor("]<-paste(substr(tmp[substr(tmp,1,7)=="factor("],7,99),")",sep="")

tmp<-paste("*",tmp,sep="")

tmp[1]<-"";

tme<-gsub(" ","",numfmt(coe[,1],5));

tme[as.numeric(tme)>0]<-paste("+",tme[as.numeric(tme)>0],sep="")

tme<-paste(tme,tmp,sep="",collapse=" ")

if (substr(tme,1,1)=="+") tme<-substr(tme,2,999);

return(paste("logit(",colvname,") = ",tme,sep=""))

}

diagrocx1<-function(d,x,xb,pngfname) {

cmp<- !is.na(d) & !is.na(x); d<-d[cmp]; x<-x[cmp]

ndL <- length(levels(factor(d)));

tt<-c(xb,format(c(sum(d),length(d)-sum(d)),nsmall=0))

if ((min(x)==max(x)) | ndL!=2) return (c(tt,rep(" ",3)))

roc0<-roc(d,x)

a.tss<-roc2xls(roc0,d)

colnames(a.tss)<-c("threshold","specificity","sensitivity","accuracy","positive-LLR","negative-LLR","diagnose-OR","N-for-diagnose","postive-pv","negative-pv","a","b","c","d")

b.tss<-a.tss[which.max(a.tss[,2]+a.tss[,3]),]

xlsfname<-paste(pngfname,"_roc.xls",sep="")

write.table(a.tss,file=xlsfname,row.names=FALSE,col.names=TRUE,sep="\t",append=FALSE,quote=FALSE)

png(paste(pngfname,"_raw.png",sep="")); rocplot(roc0,xb); dev.off()

pdf(paste(pngfname,"_raw.pdf",sep=""), width=pdfwd, height=pdfht, family="Helvetica"); rocplot(roc0,xb); dev.off()

roc1<-try(roc(d,x,smooth=bt.smooth,ci=TRUE,boot.n=bt.times,ci.alpha=0.95))

if (length(roc1)>2) {

if (bt.smooth) {

png(paste(pngfname,".png",sep="")); rocplot(roc1,xb); dev.off()

pdf(paste(pngfname,".pdf",sep=""), width=pdfwd, height=pdfht, family="Helvetica"); rocplot(roc1,xb); dev.off()

sens.ci<-try(ci.se(roc1,specificities=seq(0,1,0.05)))

if (length(sens.ci)>1) {

png(paste(pngfname,"_ci.png",sep=""))

plot(roc1,grid=TRUE,legacy.axes=TRUE,main=paste(xb, "\nAUC = ",round(roc1$auc,3)))

plot(sens.ci,type="shape",col="lightblue")

plot(sens.ci,type="bars")

dev.off()

pdf(paste(pngfname,"_ci.pdf",sep=""), width=pdfwd, height=pdfht, family="Helvetica");

plot(roc1,grid=TRUE,legacy.axes=TRUE,main=paste(xb, "\nAUC = ",round(roc1$auc,3)))

plot(sens.ci,type="shape",col="lightblue")

plot(sens.ci,type="bars")

dev.off()

}

}

aucci<-c(numfmt(c(roc1$auc,roc1$ci[c(1,3)],b.tss[1:10]),dec),b.tss[11:14])

} else {aucci<-rep(NA,17);}

return(list(c(tt,aucci),roc0))

}

diagrocx2<-function(d,x1,x1b,x2,x2b,pngfname,k) {

cmp<- !is.na(d) & !is.na(x1) & !is.na(x2); d<-d[cmp];x1<-x1[cmp];x2<-x2[cmp]

ndL <- length(levels(factor(d)));

tt1<-c(x1b,format(c(sum(d),length(d)-sum(d)),nsmall=0))

tt2<-c(x2b,format(c(sum(d),length(d)-sum(d)),nsmall=0))

if (ndL!=2) return (rbind(c(tt1,rep(" ",3)),c(tt2,rep(" ",3))))

tt<-c("Test/model","D+","D-","AUC","AUC.low","AUC.upp");

cnm<-c("threshold", "specificity", "sensitivity","accuracy","positive-LLR","negative-LLR")

cnm<-c(cnm,"diagnose-OR","N-for-diagnose","postive-pv","negative-pv","a","b","c","d")

tt<-c(tt,cnm)

if (min(x1)!=max(x1)) {

roc0<-roc(d,x1)

a.tss1<-roc2xls(roc0,d)

colnames(a.tss1)<-cnm

b.tss<-a.tss1[which.max(a.tss1[,2]+a.tss1[,3]),]

png(paste(pngfname,"_raw1.png",sep="")); rocplot(roc0,x1b); dev.off()

pdf(paste(pngfname,"_raw1.pdf",sep=""), width=pdfwd, height=pdfht, family="Helvetica"); rocplot(roc0,x1b); dev.off()

roc1<-try(roc(d,x1,smooth=bt.smooth,ci=TRUE,boot.n=bt.times,ci.alpha=0.95))

if (length(roc1)>2) {

if (bt.smooth) {

png(paste(pngfname,"_1.png",sep="")); rocplot(roc1,x1b); dev.off()

pdf(paste(pngfname,"_1.pdf",sep=""), width=pdfwd, height=pdfht, family="Helvetica"); rocplot(roc1,x1b); dev.off()

sen1.ci<-try(ci.se(roc1,specificities=seq(0,1,0.05)))

if (length(sen1.ci)>1) {

png(paste(pngfname,"_ci1.png",sep=""))

plot(roc1,xlim=c(1.0,0.0),grid=TRUE,legacy.axes=TRUE,main=paste(x1b, "\n AUC = ",round(roc1$auc,3)))

plot(sen1.ci,type="shape",col="lightblue")

plot(sen1.ci,type="bars")

dev.off()

pdf(paste(pngfname,"_ci1.pdf",sep=""), width=pdfwd, height=pdfht, family="Helvetica");

plot(roc1,xlim=c(1.0,0.0),grid=TRUE,legacy.axes=TRUE,main=paste(x1b, "\n AUC = ",round(roc1$auc,3)))

plot(sen1.ci,type="shape",col="lightblue")

plot(sen1.ci,type="bars")

dev.off()

}

}

auc1<-c(tt1,numfmt(c(roc1$auc,roc1$ci[c(1,3)],b.tss[1:10]),dec),b.tss[11:14])

} else {auc1<-rep(NA,length(tt));}

tt1=cbind(tt,auc1)

}

if (min(x2)!=max(x2)) {

roc02<-roc(d,x2)

a.tss2<-roc2xls(roc02,d)

colnames(a.tss2)<-cnm

b.tss<-a.tss2[which.max(a.tss2[,2]+a.tss2[,3]),]

png(paste(pngfname,"_raw2.png",sep="")); rocplot(roc02,x2b); dev.off()

pdf(paste(pngfname,"_raw2.pdf",sep=""), width=pdfwd, height=pdfht, family="Helvetica"); rocplot(roc02,x2b); dev.off()

roc2<-try(roc(d,x2,smooth=bt.smooth,ci=TRUE,boot.n=bt.times,ci.alpha=0.95))

if (length(roc2)>2) {

if (bt.smooth) {

png(paste(pngfname,"_2.png",sep="")); rocplot(roc2,x2b); dev.off()

pdf(paste(pngfname,"_2.pdf",sep=""), width=pdfwd, height=pdfht, family="Helvetica");rocplot(roc2,x2b); dev.off()

sen2.ci<-try(ci.se(roc2,specificities=seq(0,1,0.05)))

if (length(sen2.ci)>1) {

png(paste(pngfname,"_ci2.png",sep=""))

plot.roc(roc2,xlim=c(1.0,0.0),grid=TRUE,legacy.axes=TRUE,main=paste(x2b, "\n AUC = ",round(roc2$auc,3)))

plot(sen2.ci,type="shape",col="lightblue")

plot(sen2.ci,type="bars")

dev.off()

pdf(paste(pngfname,"_ci2.pdf",sep=""), width=pdfwd, height=pdfht, family="Helvetica");

plot.roc(roc2,xlim=c(1.0,0.0),grid=TRUE,legacy.axes=TRUE,main=paste(x2b, "\n AUC = ",round(roc2$auc,3)))

plot(sen2.ci,type="shape",col="lightblue")

plot(sen2.ci,type="bars")

dev.off()

}

}

auc2<-c(tt2,numfmt(c(roc2$auc,roc2$ci[c(1,3)],b.tss[1:10]),dec),b.tss[11:14])

} else {auc2<-rep(NA,length(tt));}

tt1=cbind(tt1,auc2)

}

if ((min(x1)!=max(x1)) & (min(x2)!=max(x2))) {

if (nrow(a.tss1)==nrow(a.tss2)) {

a.tss<-cbind(a.tss1,a.tss2)

colnames(a.tss)<-c(paste(colnames(a.tss1),"1",sep="."), paste(colnames(a.tss2),"2",sep="."))

write.table(a.tss,file=paste(pngfname,"_roc.xls",sep=""),row.names=FALSE, col.names=TRUE, sep="\t",append=FALSE,quote=FALSE)

} else {

write.table(a.tss1,file=paste(pngfname,"_roc1.xls",sep=""),row.names=FALSE, col.names=TRUE, sep="\t",append=FALSE,quote=FALSE)

write.table(a.tss2,file=paste(pngfname,"_roc2.xls",sep=""),row.names=FALSE, col.names=TRUE, sep="\t",append=FALSE,quote=FALSE)

}

if (nbg>1) {tmp.mb<-paste(bvb,bv.lb[k],sep=": ");} else {tmp.mb<-paste("Compare 2 models");}

png(paste(pngfname,"_2raw.png",sep="")); rocplot2(list(roc0,roc02),c(x1b,x2b),tmp.mb); dev.off()

pdf(paste(pngfname,"_2raw.pdf",sep=""), width=pdfwd, height=pdfht, family="Helvetica");

rocplot2(list(roc0,roc02),c(x1b,x2b),tmp.mb); dev.off()

if (length(roc1)>2 & length(roc2)>2) {

p<-roc.test(roc1,roc2,reuse.auc=FALSE,boot.n=bt.times)$p.value

ccp<-c("P(compare)",rep(" ",2),format(round(p,dec),nsmall=dec))

ccp<-c(ccp,rep(" ",length(tt)-length(ccp)))

tt1=cbind(tt1,ccp)

if (bt.times>0) {

png(paste(pngfname,"_2smooth.png",sep="")); rocplot2(list(roc1,roc2),c(x1b,x2b),tmp.mb); dev.off()

pdf(paste(pngfname,"_2smooth.pdf",sep=""), width=pdfwd, height=pdfht, family="Helvetica");

rocplot2(list(roc1,roc2),c(x1b,x2b),tmp.mb); dev.off()

}

}

}

if (!is.matrix(tt1)) {

return(rep(" ",length(tt)));

} else {

if (ncol(tt1)>1) {return(tt1[,-1])} else {return(rep(" ",length(tt)))}

}

}

plotnomogram <- function(tmp.nom) {

tmp.nom.x<-tmp.nom[length(tmp.nom)][[1]]$x

x.mid<-(max(tmp.nom.x)+min(tmp.nom.x))/2

x.mid.dis<-abs(tmp.nom.x-x.mid)

x.setblank<-(1:length(tmp.nom.x))[-c(1,which(x.mid.dis==min(x.mid.dis)),length(tmp.nom.x))]

tmp.nom[length(tmp.nom)][[1]]$fat[x.setblank]<-""

lbl.nchar<-max(nchar(c(names(tmp.nom),"linear.predictor")))

lblfac<-0.2

if (lbl.nchar>20) lblfac<-0.3

if (lbl.nchar>30) lblfac<-0.4

plot(tmp.nom,xfrac=lblfac);

}

mdl2oddsratio<-function(mdlobj, rr="OR") {

tmps <- summary(mdlobj)$coefficients

tmpc <- cbind(tmps[,1],tmps[,1]-1.96*tmps[,2],tmps[,1]+1.96*tmps[,2])

tmpor<- cbind(exp(tmpc),tmps[,4])

colnames(tmpor) <- c(rr,"Low 95%CI","High 95%CI", "P value")

rownames(tmpor) <- rownames(tmps)

return(tmpor)

}

mat2htmltable<-function(mat, rcname=FALSE) {

if (rcname) mat<-cbind(c("",rownames(mat)),rbind(colnames(mat),mat))

t1<- apply(mat,1,function(z) paste(z,collapse="</td><td>"))

t2<- paste("<tr><td>",t1,"</td></tr>")

return(paste(t2,collapse=" "))

}

vlabelN<-(substr(vlabel,1,1)==" ");

vlabelZ<-vlabel[vlabelN];vlabelV<-vlabel[!vlabelN]

vnameV<-vname[!vlabelN];vnameZ<-vname[vlabelN]

if (is.na(parm[1])) parm[1]<-0

bt.smooth<-(parm[1]==1); bt.times<-500;

if (is.na(parm[2])) {

study.type <- "cohort";

} else {

if (parm[2]>=1) parm[2] <- parm[2]/100

if (parm[2]>0) {study.type<-"case-control"; population.r <- parm[2];} else {study.type="cohort";}

}

if (is.na(avname[1]) & par1==3) par1==2;

if (!is.na(bvar)) {

bvb<-vlabelV[match(bvar,vnameV)]; if (is.na(bvb)) bvb<-bvar;

bv.lv<-levels(factor(bv));

bv.lb<-vlabelZ[match(paste(bvar,bv.lv,sep="."),vnameZ)]

bv.lb[is.na(bv.lb)]<-bv.lv[is.na(bv.lb)]

nbg<-length(bv.lv);

} else {bv<-rep(1,nrow(WD)); bv.lv<-rep(1,nrow(WD)); nbg<-1; bvb<-"ALL";}

nx=ncol(xv); xb<-vlabelV[match(xvname,vnameV)]; xb[is.na(xb)]<-xvname[is.na(xb)];

colv.lv<-levels(factor(colv))

yb<-vlabelV[match(colvname,vnameV)]; if (is.na(yb)) yb<-colvname;

ylb<-c("0","1")

if (length(colv.lv)==2 & colv.lv[1]==0 & colv.lv[2]==1) {

yv<-colv;

} else {

if (length(colv.lv)>=2) {

yv<-(colv==colv.lv[length(colv.lv)])*1; yv[is.na(colv)]<-NA;

ylb<-c("0",paste(colvname,"=",colv.lv[length(colv.lv)],sep=""))

} else {par1==0;}

}

ofname1<-ofname;

tmp.ss<-rep(NA,13); if (nbg>1) tmp.ss<-rep(NA,14)

tmp.roc<-rep(NA,20)

dec<-4; rocc<-list(NA)

sink(paste(ofname1,".txt",sep=""))

tmp.fml<-NULL

w.00<-""

tmp1<-c("Test","1","0","ROC area(AUC)","95%CI low","95%CI upp","Best threshold","Specificity","Sensitivity")

tmp1<-c(tmp1,"Accuracy","Positive-LR","Negative-LR","Diagnose-OR","N-for-diagnose","Postive-pv","Negative-pv","a","b","c","d")

for (k in (1:nbg)) {

ncxplot<-0

if (nbg>1) {

print(paste(bvb,"=",bv.lb[k]))

w.00<-c(w.00,"</br>", paste(bvb,"=",bv.lb[k]), "</br></br>")

}

if (par1==1) {

dcas <- list()

ndca <- 0

dcaname <- NULL

for (i in (1:nx)) {

x.tmp<-xv[bv==bv.lv[k],i]; y.tmp<-yv[bv==bv.lv[k]]

cmp<-(!is.na(x.tmp) & !is.na(y.tmp))

x.tmp<-x.tmp[cmp]; y.tmp<-y.tmp[cmp]; WDTMP <- as.data.frame(cbind(y.tmp,x.tmp))

singleChk.x<-(max(x.tmp)!=min(x.tmp))

if (length(levels(factor(y.tmp)))==2 & singleChk.x) {

pngf<-paste(ofname1,xvname[i],sep="_")

if (nbg>1) pngf<-paste(pngf,bv.lv[k],sep="_")

if (xlv[i]==0) {

tmp.xb<-xb[i]; if (nbg>1) tmp.xb<-paste(tmp.xb," (",bvb,"=",bv.lb[k],")",sep="")

tmp<-diagrocx1(y.tmp,x.tmp,tmp.xb,pngf)

tmp.roc<-rbind(tmp.roc,tmp[[1]])

ncxplot<-ncxplot+1

rocc[[ncxplot]]<-tmp[[2]]

if (ncxplot==1) {xxb<-tmp.xb;} else {xxb<-c(xxb,tmp.xb);}

ndca <- ndca + 1

dcas[[ndca]] <- decision_curve(y.tmp~x.tmp, data=WDTMP, study.design="cohort", policy="opt-in", bootstraps = 50)

write.table(dcas[[ndca]]$derived.data,file=paste(pngf,xvname[i],"dca.xls",sep="_"),col.names=TRUE,row.names=FALSE,sep="\t")

if (is.null(dcaname)) {dcaname <- xvname[i];} else {dcaname<-c(dcaname, xvname[i]);}

} else {

xi.lv<-levels(factor(x.tmp))

tmp.xlb<-vlabelZ[match(paste(xvname[i],xi.lv,sep="."),vnameZ)]

tmp.xlb[is.na(tmp.xlb)]<-xi.lv[is.na(tmp.xlb)]

tmp<-diagss(y.tmp,x.tmp,xb[i],tmp.xlb,ylb,pngf)

if (nbg>1) tmp<-cbind(bv.lb[k],tmp)

tmp.ss<-rbind(tmp.ss,tmp)

}

}

rm(WDTMP)

}

if (ncxplot>1) {

pngf<-ofname1; if (nbg>1) pngf<-paste(pngf,bv.lv[k],sep="_")

if (nbg>1) {tmp.mb<-paste(bvb,bv.lb[k],sep=": ");} else {tmp.mb<-paste("ROC curve for", yb);}

png(paste(pngf,"_rocs.png",sep=""),width=640,height=640); rocplot2(rocc,xxb,tmp.mb); dev.off()

pdf(paste(pngf,"_rocs.pdf",sep=""),width=pdfwd, height=pdfht, family="Helvetica");rocplot2(rocc,xxb,tmp.mb); dev.off()

}

if (is.matrix(tmp.ss)) {

tmp0<-c("Test","1","0","Likelihood ratio(LR)","Sensitivity","Specificity","Positive pv","Negative pv","Accuracy","Positive-LR","Negative-LR","Diagnosis OR","Number for diagnose")

if (nbg>1) tmp0<-c(bvb,tmp0)

tmp.ss<-rbind(tmp0,tmp.ss[-1,])

w.00<-c(w.00,"</br>Sensitivity and Specificity for diagnositic test</br><table border=3>",mat2htmltable(tmp.ss),"</table>")

}

if (is.matrix(tmp.roc)) {

tmp.roc<-rbind(tmp1,tmp.roc[-1,])

tmp.roc1<-tmp.roc[,(1:9)]

tmp.roc2<-tmp.roc[,-(2:6)]

w.00<-c(w.00,"</br>ROC analysis for continuous predictor</br><table border=3>",mat2htmltable(tmp.roc1),"</table>")

w.00<-c(w.00,"</br>Best threshold analysis</br><table border=3>",mat2htmltable(tmp.roc2),"</table>")

}

if (ndca>0) {

png(paste(pngf,"_dca.png",sep=""),width=960,height=840);

if (study.type=="cohort") {

plot_decision_curve(dcas, curve.names = dcaname, col = (2:(ndca+1)), confidence.intervals = FALSE)

} else {

plot_decision_curve(dcas, curve.names = dcaname, col = (2:(ndca+1)), confidence.intervals = FALSE,

study.design = "case-control", population.prevalence = population.r)

}

dev.off()

pdf(paste(pngf,"_dca.pdf",sep=""),width=pdfwd*1.5, height=pdfht*1.5, family="Helvetica");

if (study.type=="cohort") {

plot_decision_curve(dcas, curve.names = dcaname, col = (2:(ndca+1)), confidence.intervals = FALSE)

} else {

plot_decision_curve(dcas, curve.names = dcaname, col = (2:(ndca+1)), confidence.intervals = FALSE,

study.design = "case-control", population.prevalence = population.r)

}

dev.off()

}

}

if (par1==2) {

WD0<-cbind(yv,xv); colnames(WD0)<-c(colvname,xvname);

if (nbg>1) WD0<-WD0[bv==bv.lv[k],]

cmp<-(apply(is.na(WD0),1,sum)==0)

WD0<-as.data.frame(WD0[cmp,])

if (length(levels(factor(WD0[,1])))==2) {

xv1.1<-xvname

xv1.1[xlv>2]<-paste("factor(",xv1.1[xlv>2],")",sep="")

singleChk<- apply(cbind(WD0[,xvname],1),2,function(x) return(max(x,na.rm=TRUE)-min(x,na.rm=TRUE)!=0))

fml.1<-paste(colvname,"~",paste(xv1.1[singleChk],collapse="+"))

tmp.glm<-try(glm(formula(fml.1),family=binomial(link="logit"),data=WD0))

pngf<-paste(ofname1,"model",sep="_")

if (nbg>1) pngf<-paste(pngf,bv.lv[k],sep="_")

if (substr(tmp.glm[[1]][1],1,5)!="Error") {

print(summary(tmp.glm)); print(mdl2oddsratio(tmp.glm))

tmp.lrm<-try(lrm(formula(fml.1),data=WD0))

if (substr(tmp.lrm[[1]][1],1,5)!="Error") {

tmp.dst<-datadist(WD0)

options(datadist='tmp.dst')

tmp.nom<-nomogram(tmp.lrm, fun=function(x)1/(1+exp(-x)),funlabel=yb)

print("Nomogram"); print(tmp.nom)

png(paste(pngf,"_nom.png",sep=""),width=960,height=840); plotnomogram(tmp.nom); dev.off()

pdf(paste(pngf,"_nom.pdf",sep=""),width=pdfwd*1.5, height=pdfht*1.5, family="Helvetica");plotnomogram(tmp.nom); dev.off()

}

x.tmp<-predict(tmp.glm)

y.tmp<-WD0[,1]

tmp.xb<-"Model";

tmpi<-glm2formula(tmp.glm)

if (nbg>1) {

tmp.xb<-paste(tmp.xb," (",bvb,"=",bv.lb[k],")",sep="")

tmpi<-paste("</br>",bvb,"=",bv.lb[k],":</br>",tmpi)

}

tmp.fml<-c(tmp.fml,tmpi)

tmp<-diagrocx1(y.tmp,x.tmp,tmp.xb,pngf)

tmp.roc<-cbind(tmp.roc,tmp[[1]])

dca0 <- decision_curve(formula(fml.1), data=WD0, study.design="cohort", policy="opt-in", bootstraps = 50)

png(paste(pngf,"_dca.png",sep=""),width=960,height=840);

if (study.type=="cohort") {

plot_decision_curve(dca0, curve.names="Model", confidence.intervals = FALSE, col="red")

} else {

plot_decision_curve(dca0, curve.names="Model", confidence.intervals = FALSE, col="red",

study.design = "case-control", population.prevalence = population.r)

}

dev.off()

pdf(paste(pngf,"_dca.pdf",sep=""),width=pdfwd*1.5, height=pdfht*1.5, family="Helvetica");

if (study.type=="cohort") {

plot_decision_curve(dca0, curve.names="Model", confidence.intervals = FALSE, col="red")

} else {

plot_decision_curve(dca0, curve.names="Model", confidence.intervals = FALSE, col="red",

study.design = "case-control", population.prevalence = population.r)

}

dev.off()

write.table(dca0$derived.data,file=paste(pngf,"_dca.xls",sep=""),col.names=TRUE,row.names=FALSE,sep="\t")

} else {tmp.roc<-"Model error!";}

rm(WD0)

}

if (is.matrix(tmp.roc)) {

tmp.roc<-cbind(tmp1,tmp.roc[,-1])

} else {w.00<-c(w.00,"</br>",tmp.roc);}

}

if (par1==3) {

WD0<-cbind(yv,xv,av); colnames(WD0)<-c(colvname,xvname,avname)

if (nbg>1) WD0<-WD0[bv==bv.lv[k],]

cmp<-(apply(is.na(WD0),1,sum)==0)

WD0<-as.data.frame(WD0[cmp,])

if (length(levels(factor(WD0[,1])))==2) {

xv1<-xvname

xv1[xlv!=0]<-paste("factor(",xv1[xlv!=0],")",sep="")

singleChk.xv1 <- apply(cbind(WD0[,xvname],1),2,function(x) return(max(x)-min(x)!=0))

xv1<- xv1[singleChk.xv1];

fml<-paste(colvname,"~",paste(xv1,collapse="+"))

tmp.glm<-try(glm(formula(fml),family=binomial(link="logit"),data=WD0))

if (substr(tmp.glm[[1]][1],1,5)!="Error") {

or.tb1<- numfmt(mdl2oddsratio(tmp.glm),4)

print(summary(tmp.glm)); print(or.tb1)

x.tmp<-predict(tmp.glm)

y.tmp<-WD0[,1]

av1<-avname

av1[alv!=0]<-paste("factor(",av1[alv!=0],")",sep="")

singleChk.av1 <- apply(cbind(WD0[,avname],1),2,function(x) return(max(x)-min(x)!=0))

av1<- av1[singleChk.av1];

fml2<-paste(colvname,"~",paste(av1,collapse="+"))

tmp.glm2<-try(glm(formula(fml2),family=binomial(link="logit"),data=WD0))

if (substr(tmp.glm2[[1]][1],1,5)!="Error") {

or.tb2<- numfmt(mdl2oddsratio(tmp.glm2),4)

print(summary(tmp.glm2)); print(or.tb2)

x.tmp2<-predict(tmp.glm2)

pngf<-paste(ofname1,"model2",sep="_")

if (nbg>1) pngf<-paste(pngf,bv.lv[k],sep="_")

tmp.x1b<-"Model1"; if (nbg>1) tmp.x1b<-paste(tmp.x1b,"(",bvb,"=",bv.lb[k],")",sep="")

tmp.x2b<-"Model2"; if (nbg>1) tmp.x2b<-paste(tmp.x2b,"(",bvb,"=",bv.lb[k],")",sep="")

tmp.fml<-glm2formula(tmp.glm)

tmp.fml2<-glm2formula(tmp.glm2)

tmp.roc<-cbind(tmp.roc,diagrocx2(y.tmp,x.tmp,tmp.x1b,x.tmp2,tmp.x2b,pngf,k))

impr<-improveProb(x.tmp>=as.numeric(tmp.roc[7,2]), x.tmp2>=as.numeric(tmp.roc[7,3]), y.tmp)

print(impr)

w.nrp<-c("Increase for events (1)", "Increase for non-events (2)", "Decrease for events (3)", "Decrease for non-events (4)")

w.nrp<-cbind(w.nrp, numfmt(unlist(impr[c("pup.ev","pup.ne","pdown.ev","pdown.ne")]), dec))

nri<-matrix(unlist(impr[c("nri", "nri.ev", "nri.ne", "idi", "se.nri", "se.nri.ev", "se.nri.ne", "se.idi")]),ncol=2)

nri<-cbind(nri, nri[,1]/nri[,2])

nri<-cbind(nri, 2-2*pnorm(abs(nri[,3])))

nri<-cbind(nri, nri[,1]-1.96*nri[,2], nri[,1]+1.96*nri[,2])

w.nri<-numfmt(nri,dec)

w.nri[,4]<-pvformat(nri[,4],4)

w.nri<-rbind(c("Estimate","SE","Z","P value","95% Lower","95% Upper"), w.nri)

w.nri<-cbind(c("","NRI (1-3+4-2)","NRI for events (1-3)","NRI for non-events (4-2)","IDI"),w.nri)

dca1 <- decision_curve(formula(fml), data=WD0, study.design="cohort", policy="opt-in", bootstraps = 50)

dca2 <- decision_curve(formula(fml2), data=WD0, study.design="cohort", policy="opt-in", bootstraps = 50)

png(paste(pngf,"_dca.png",sep=""),width=960,height=840);

if (study.type=="cohort") {

plot_decision_curve(list(dca1, dca2), curve.names = c("Model 1", "Model 2"), col = c("blue", "red"), confidence.intervals = FALSE)

} else {

plot_decision_curve(list(dca1, dca2), curve.names = c("Model 1", "Model 2"), col = c("blue", "red"), confidence.intervals = FALSE,

study.design = "case-control", population.prevalence = population.r)

}

dev.off()

pdf(paste(pngf,"_dca.pdf",sep=""),width=pdfwd*1.5, height=pdfht*1.5, family="Helvetica");

if (study.type=="cohort") {

plot_decision_curve(list(dca1, dca2), curve.names = c("Model 1", "Model 2"), col = c("blue", "red"), confidence.intervals = FALSE)

} else {

plot_decision_curve(list(dca1, dca2), curve.names = c("Model 1", "Model 2"), col = c("blue", "red"), confidence.intervals = FALSE,

study.design = "case-control", population.prevalence = population.r)

}

dev.off()

write.table(dca1$derived.data,file=paste(pngf,"_dca1.xls",sep=""),col.names=TRUE,row.names=FALSE,sep="\t")

write.table(dca2$derived.data,file=paste(pngf,"_dca2.xls",sep=""),col.names=TRUE,row.names=FALSE,sep="\t")

} else {tmp.roc<-"Model 2 error!";}

} else {tmp.roc<-"Model 1 error!";}

rm(WD0)

}

if (is.matrix(tmp.roc)) {

tmp.roc<-cbind(tmp1,tmp.roc[,-1])

w.00<-c(w.00,"</br>Predictive model:</br>","Model 1：",tmp.fml,"</br>","Model 2: ",tmp.fml2)

w.00<-c(w.00,"</br></br>Compare 2 predictive models (ROC Curve)</br><table border=3>",mat2htmltable(tmp.roc),"</table>")

w.00<-c(w.00,"</br></br>Net Reclassification Index, Integrated Discrimination Index")

w.00<-c(w.00,"</br><table border=3>",mat2htmltable(w.nrp),"</table>")

w.00<-c(w.00,"</br><table border=3>",mat2htmltable(w.nri),"</table>")

w.00<-c(w.00,"</br>Model 1</br><table border=3>",mat2htmltable(or.tb1,TRUE),"</table>")

w.00<-c(w.00,"</br>Model 2</br><table border=3>",mat2htmltable(or.tb2,TRUE),"</table>")

} else {w.00<-c(w.00,"</br>",tmp.roc);}

}

}

sink()

w<-c("<html><head>","<meta http-equiv=\"Content-Type\" content=\"text/html\" charset=\"gb2312\" /></head><body>")

w<-c(w,"<h2>",title,"</h2>")

w<-c(w,"</br>Outcome variable:", yb, "</br>")

if (par1==2) {

w <-c(w,"</br>Predictive model:</br>",tmp.fml)

tmp1<-c("Test items","1","0","ROC area(AUC)","95% Interval lower limit","95% Interval upper limit","Optimal threshold","Specificity","Sensitivity")

tmp1<-c(tmp1,"Accuracy","Positive-LR","Negative-LR","Diagnosis OR","Number for diagnose","Positive pv","Negative pv","a","b","c","d");

tmp.roc<-cbind(tmp1,tmp.roc[,-1])

w<-c(w,"</br></br>Model ROC curve and best threshold analysis</br><table border=3>",mat2htmltable(tmp.roc),"</table>")

} else {

w<-c(w,w.00)

w<-c(w,"</br>Sensitivity; Specificity; Positive pv; Negative pv; Accuracy; Positive-LR; Negative-LR; Diagnosis OR; Number for diagnose</br>")

}

if (bt.smooth) w<-c(w,paste("</br>AUC Confidence interval and significance test using non-parametric repeated sampling method (Bootstrap resampling times=",bt.times,")"))

w<-c(w,"</br></br> The optimal threshold is taken as the cut-off value with the maximum sensitivity + specificity. The sensitivity specificity corresponding to each cut-off point is saved in the ROC output file (.xls) ")

w<-c(w,"</body></html>")

fileConn<-file(paste(ofname1,".htm",sep="")); writeLines(w, fileConn)

**S1.2 ROC by using Bootstrap resampling in different genders for different IR surrogates to predict HU**

R.Version4RUN<-343;

R.LibLocation <- "C:/Users/Administrator/AppData/Roaming/EmpowerRCH/R343/library"

Sys.setlocale("LC_TIME", "C")

library(doBy,lib.loc=R.LibLocation)

library(plotrix,lib.loc=R.LibLocation)

library(stringi,lib.loc=R.LibLocation)

library(stringr,lib.loc=R.LibLocation)

library(survival,lib.loc=R.LibLocation)

library(rms,lib.loc=R.LibLocation)

library(nnet,lib.loc=R.LibLocation)

library(car,lib.loc=R.LibLocation)

library(mgcv,lib.loc=R.LibLocation)

pdfwd<-6; pdfht<-6

setwd("F:/data_process/IR.UA2/PROJ29_25_tbl")

load("F:/data_process/IR.UA2/wt.Rdata")

if (length(which(ls()=="EmpowerStatsR"))==0) EmpowerStatsR<-get(ls()[1])

names(EmpowerStatsR)<-toupper(names(EmpowerStatsR))

rankvar <- function(var, num) {

qprobs <- 1/num

if (num>2) {for (i in (2:(num-1))) {qprobs <- c(qprobs, 1/num * i) }}

outvar <- rep(0, times=length(var))

outvar[is.na(var)] <- NA

cutpoints <- quantile(var,probs=qprobs, na.rm=TRUE)

for (k in (1:length(cutpoints))) {outvar[var>=cutpoints[k]] <- k}

tmp<-c(min(var,na.rm=TRUE),cutpoints,max(var,na.rm=TRUE))

names(tmp)<-c("Min",names(cutpoints),"Max")

print(tmp)

return(outvar)

}

recodevar <- function (var,oldcode,newcode) {

tmp.v <- var

nc.tmp <- length(oldcode)

for (i in (1:nc.tmp)) {tmp.v[(var==oldcode[i])]=newcode[i]}

if (is.factor(tmp.v)) {tmp.v1<-as.numeric(as.character(tmp.v))} else {tmp.v1<-as.numeric(tmp.v)}

rm(tmp.v); return(tmp.v1)

}

attach(EmpowerStatsR)

sink("F:/data_process/IR.UA2/datastep/PROJ29_datastep.lst")

print("Create new variable: POPSLT.CRITERIA1")

POPSLT.CRITERIA1<-ifelse((!is.na(X8) & !is.na(X14)),1,0)

EmpowerStatsR<-cbind(EmpowerStatsR,POPSLT.CRITERIA1)

POPSLT.STEP1<-POPSLT.CRITERIA1

print("Create new variable: POPSLT.CRITERIA2")

POPSLT.CRITERIA2<-ifelse((!is.na(X8) & !is.na(X14) & (!is.na(X20) & (X20!=2))),1,0)

EmpowerStatsR<-cbind(EmpowerStatsR,POPSLT.CRITERIA2)

print("Creating new variable: POPSLT.STEP2")

POPSLT.STEP2<-ifelse((POPSLT.STEP1==1 & POPSLT.CRITERIA2==1),1,0)

EmpowerStatsR<-cbind(EmpowerStatsR,POPSLT.STEP2)

print("Create new variable: POPSLT.CRITERIA3")

POPSLT.CRITERIA3<-ifelse((!is.na(X8) & !is.na(X14) & (!is.na(X20) & (X20!=2)) & (X3<10.5)),1,0)

EmpowerStatsR<-cbind(EmpowerStatsR,POPSLT.CRITERIA3)

print("Creating new variable: POPSLT.STEP3")

POPSLT.STEP3<-ifelse((POPSLT.STEP2==1 & POPSLT.CRITERIA3==1),1,0)

EmpowerStatsR<-cbind(EmpowerStatsR,POPSLT.STEP3)

print("Creating new variable: HYPER.UA1")

HYPER.UA1<-rep(NA,times=nrow(EmpowerStatsR))

tmp<- (is.na(HYPER.UA1) & (X5==1 & X17>=7))

tmp[is.na(tmp)]<-FALSE

HYPER.UA1[tmp]<-1

tmp<- (is.na(HYPER.UA1) & (X5==2 & X17>=6))

tmp[is.na(tmp)]<-FALSE

HYPER.UA1[tmp]<-1

tmp<-(is.na(HYPER.UA1))

HYPER.UA1[tmp]<-0

EmpowerStatsR<-cbind(EmpowerStatsR,HYPER.UA1)

print("Creating new variable: TYG.BMI")

TYG.BMI<- X3*X8

EmpowerStatsR<-cbind(EmpowerStatsR,TYG.BMI)

print("Creating new variable: TG.HDL")

TG.HDL <- X2/X14

EmpowerStatsR<-cbind(EmpowerStatsR,TG.HDL)

print("Creating new variable: MET1")

MET1 <- log(2*X15+X2)*X8

EmpowerStatsR<-cbind(EmpowerStatsR,MET1)

print("Creating new variable: MET2")

MET2 <- log(X14)

EmpowerStatsR<-cbind(EmpowerStatsR,MET2)

print("Creating new variable: X3.Q4")

X3.Q4<- rankvar(X3,4)

EmpowerStatsR<-cbind(EmpowerStatsR,X3.Q4)

print("Creating new variable: TYG.BMI.Q4")

TYG.BMI.Q4<- rankvar(TYG.BMI,4)

EmpowerStatsR<-cbind(EmpowerStatsR,TYG.BMI.Q4)

print("Creating new variable: TG.HDL.Q4")

TG.HDL.Q4<- rankvar(TG.HDL,4)

EmpowerStatsR<-cbind(EmpowerStatsR,TG.HDL.Q4)

print("Creating new variable: MET.IR")

MET.IR <- MET1/MET2

EmpowerStatsR<-cbind(EmpowerStatsR,MET.IR)

print("Creating new variable: MET.IR.Q4")

MET.IR.Q4<- rankvar(MET.IR,4)

EmpowerStatsR<-cbind(EmpowerStatsR,MET.IR.Q4)

print("Creating new variable: X17.CS")

X17.CS<- 0+(X17<6)

X17.CS[is.na(X17)]<-NA

EmpowerStatsR<-cbind(EmpowerStatsR,X17.CS)

print("Creating new variable: X17.CS.NEW")

X17.CS.NEW<- recodevar(X17.CS,c(0,1),c(1,0))

EmpowerStatsR<-cbind(EmpowerStatsR,X17.CS.NEW)

print("Creating new variable: X3.CS")

X3.CS<- 0+(X3>=8.44)

X3.CS[is.na(X3)]<-NA

EmpowerStatsR<-cbind(EmpowerStatsR,X3.CS)

print("Creating new variable: TYG.BMI.CS")

TYG.BMI.CS<- 0+(TYG.BMI>=224.16)

TYG.BMI.CS[is.na(TYG.BMI)]<-NA

EmpowerStatsR<-cbind(EmpowerStatsR,TYG.BMI.CS)

print("Creating new variable: TG.HDL.CS")

TG.HDL.CS<- 0+(TG.HDL>=1.77)

TG.HDL.CS[is.na(TG.HDL)]<-NA

EmpowerStatsR<-cbind(EmpowerStatsR,TG.HDL.CS)

print("Creating new variable: MET.IR.CS")

MET.IR.CS<- 0+(MET.IR>=39.52)

MET.IR.CS[is.na(MET.IR)]<-NA

EmpowerStatsR<-cbind(EmpowerStatsR,MET.IR.CS)

print("Creating new variable: X3.CS1")

X3.CS1<- 0+(X3>=8.48)

X3.CS1[is.na(X3)]<-NA

EmpowerStatsR<-cbind(EmpowerStatsR,X3.CS1)

print("Creating new variable: TYG.BMI.CS1")

TYG.BMI.CS1<- 0+(TYG.BMI>=231.26)

TYG.BMI.CS1[is.na(TYG.BMI)]<-NA

EmpowerStatsR<-cbind(EmpowerStatsR,TYG.BMI.CS1)

print("Creating new variable: TG.HDL.CS1")

TG.HDL.CS1<- 0+(TG.HDL>=1.78)

TG.HDL.CS1[is.na(TG.HDL)]<-NA

EmpowerStatsR<-cbind(EmpowerStatsR,TG.HDL.CS1)

print("Creating new variable: MET.IR.CS1")

MET.IR.CS1<- 0+(MET.IR>=39.52)

MET.IR.CS1[is.na(MET.IR)]<-NA

EmpowerStatsR<-cbind(EmpowerStatsR,MET.IR.CS1)

print("Creating new variable: X3.CS2")

X3.CS2<- 0+(X3>=8.44)

X3.CS2[is.na(X3)]<-NA

EmpowerStatsR<-cbind(EmpowerStatsR,X3.CS2)

print("Creating new variable: TYG.BMI.CS2")

TYG.BMI.CS2<- 0+(TYG.BMI>=241)

TYG.BMI.CS2[is.na(TYG.BMI)]<-NA

EmpowerStatsR<-cbind(EmpowerStatsR,TYG.BMI.CS2)

print("Creating new variable: TG.HDL.CS2")

TG.HDL.CS2<- 0+(TG.HDL>=1.77)

TG.HDL.CS2[is.na(TG.HDL)]<-NA

EmpowerStatsR<-cbind(EmpowerStatsR,TG.HDL.CS2)

print("Creating new variable: MET.IR.CS2")

MET.IR.CS2<- 0+(MET.IR>=42.5)

MET.IR.CS2[is.na(MET.IR)]<-NA

EmpowerStatsR<-cbind(EmpowerStatsR,MET.IR.CS2)

rm(HYPER.UA1,TYG.BMI,TG.HDL,MET1,MET2,X3.Q4,TYG.BMI.Q4,TG.HDL.Q4,MET.IR,MET.IR.Q4,X17.CS,X17.CS.NEW,X3.CS,TYG.BMI.CS,TG.HDL.CS,MET.IR.CS,X3.CS1,TYG.BMI.CS1,TG.HDL.CS1,MET.IR.CS1,X3.CS2,TYG.BMI.CS2,TG.HDL.CS2,MET.IR.CS2)

rm(POPSLT.CRITERIA1,POPSLT.STEP1,POPSLT.CRITERIA2,POPSLT.STEP2,POPSLT.CRITERIA3,POPSLT.STEP3)

detach(EmpowerStatsR)

sink()

vname<-c("_N_","_STAT_","_TOTAL_","X1","X2","X3","X4")

vlabel<-c("Sample size(%)","Statistics","Total","seqn","TG","TyG","age")

vname<-c(vname,"X5","X6","X7","X8","X9","X10")

vlabel<-c(vlabel,"sex","eth","nhs_wt","BMI","smoke","drink")

vname<-c(vname,"X11","X12","X13","X14","X15")

vlabel<-c(vlabel,"PA_total_MET","TC","LDL-C","HDL-C","FPG")

vname<-c(vname,"X16","X17","X18","X19","X20","X20.1","X20.2","X20.3")

vlabel<-c(vlabel,"eGFR","UA","sbp","dbp","DM"," no"," yes"," pre")

vname<-c(vname,"X21","X21.1","X21.2","X22","X22.1","X22.2")

vlabel<-c(vlabel,"Hyperlipidemia"," no"," yes","Hypertension"," no"," yes")

vname<-c(vname,"X23","X23.1","X23.2","X24","X24.1","X24.2")

vlabel<-c(vlabel,"Diabetic"," no"," yes","Hyperlipidemic"," no"," yes")

vname<-c(vname,"X25","X25.1","X25.2","X26","X26.1","X26.2","X26.3")

vlabel<-c(vlabel,"Hypertensive"," no"," yes","sdmvpsu"," 1"," 2"," 3")

vname<-c(vname,"X27","X28","X29","X29.1","X29.2","X29.3")

vlabel<-c(vlabel,"sdmvstra","wtmec2yr","education"," less than high school"," high school"," more than high school")

vname<-c(vname,"HYPER.UA1","HYPER.UA1.0","HYPER.UA1.1","TYG.BMI")

vlabel<-c(vlabel,"HYPER.UA1"," 0"," 1","TyG * BMI")

vname<-c(vname,"TG.HDL","MET1","MET2","X3.Q4","X3.Q4.0","X3.Q4.1","X3.Q4.2","X3.Q4.3")

vlabel<-c(vlabel,"TG.HDL","MET1","MET2","TyG Quartered"," Q1"," Q2"," Q3"," Q4")

vname<-c(vname,"TYG.BMI.Q4","TYG.BMI.Q4.0","TYG.BMI.Q4.1","TYG.BMI.Q4.2","TYG.BMI.Q4.3")

vlabel<-c(vlabel,"TyG * BMI Quartered"," Q1"," Q2"," Q3"," Q4")

vname<-c(vname,"TG.HDL.Q4","TG.HDL.Q4.0","TG.HDL.Q4.1","TG.HDL.Q4.2","TG.HDL.Q4.3")

vlabel<-c(vlabel,"TG.HDL Quartered"," Q1"," Q2"," Q3"," Q4")

vname<-c(vname,"MET.IR","MET.IR.Q4","MET.IR.Q4.0","MET.IR.Q4.1","MET.IR.Q4.2","MET.IR.Q4.3")

vlabel<-c(vlabel,"MET.IR","MET.IR Quartered"," Q1"," Q2"," Q3"," Q4")

vname<-c(vname,"X17.CS","X17.CS.0","X17.CS.1","X17.CS.NEW","X17.CS.NEW.0","X17.CS.NEW.1")

vlabel<-c(vlabel,"UA group"," >=6"," <6","UA group NEW"," 0"," 1")

vname<-c(vname,"X3.CS","X3.CS.0","X3.CS.1","TYG.BMI.CS","TYG.BMI.CS.0","TYG.BMI.CS.1")

vlabel<-c(vlabel,"TyG group"," <8.44"," >=8.44","TyG * BMI group"," <224.16"," >=224.16")

vname<-c(vname,"TG.HDL.CS","TG.HDL.CS.0","TG.HDL.CS.1","MET.IR.CS","MET.IR.CS.0","MET.IR.CS.1")

vlabel<-c(vlabel,"TG.HDL group"," <1.77"," >=1.77","MET.IR group"," <39.52"," >=39.52")

vname<-c(vname,"X3.CS1","X3.CS1.0","X3.CS1.1","TYG.BMI.CS1","TYG.BMI.CS1.0","TYG.BMI.CS1.1")

vlabel<-c(vlabel,"TyG group"," <8.48"," >=8.48","TyG * BMI group"," <231.26"," >=231.26")

vname<-c(vname,"TG.HDL.CS1","TG.HDL.CS1.0","TG.HDL.CS1.1")

vlabel<-c(vlabel,"TG.HDL group"," <1.78"," >=1.78")

vname<-c(vname,"MET.IR.CS1","MET.IR.CS1.0","MET.IR.CS1.1")

vlabel<-c(vlabel,"MET.IR group"," <39.52"," >=39.52")

vname<-c(vname,"X3.CS2","X3.CS2.0","X3.CS2.1","TYG.BMI.CS2","TYG.BMI.CS2.0","TYG.BMI.CS2.1")

vlabel<-c(vlabel,"TyG group"," <8.44"," >=8.44","TyG * BMI group"," <241"," >=241")

vname<-c(vname,"TG.HDL.CS2","TG.HDL.CS2.0","TG.HDL.CS2.1")

vlabel<-c(vlabel,"TG.HDL group"," <1.77"," >=1.77")

vname<-c(vname,"MET.IR.CS2","MET.IR.CS2.0","MET.IR.CS2.1")

vlabel<-c(vlabel,"MET.IR group"," <42.5"," >=42.5")

slt.vname<-c()

library(pROC,lib.loc=R.LibLocation)

library(survivalROC,lib.loc=R.LibLocation)

library(plyr,lib.loc=R.LibLocation)

library(rms,lib.loc=R.LibLocation)

library(rmda,lib.loc=R.LibLocation)

library(Hmisc,lib.loc=R.LibLocation)

ofname<-"PROJ29_25_tbl";

attach(EmpowerStatsR);WD<-EmpowerStatsR[(POPSLT.CRITERIA2==1),];detach(EmpowerStatsR);

wd.subset<-paste("Use subset of data:","POPSLT.CRITERIA2==1");svy.DSN.YN <- FALSE;

weights<-1;weights.var <- NA;

WD<-cbind(WD,weights); WD<-WD[!is.na(weights),];

title<-"Diagnostic test with ROC analysis - gender-specific - resampling";

attach(WD)

subjvname<-NA;

xv<-cbind(X3,TYG.BMI,TG.HDL,MET.IR);

xvname<-c('X3','TYG.BMI','TG.HDL','MET.IR');

xvar<-c('X3','TYG_BMI','TG_HDL','MET_IR');

xlv<-c(0,0,0,0);

sxf<-NA;

svname<-NA; sv<-NA; slv<-NA;

av<-NA; avname<-NA; avlbl<-NA; nadj<-0; alv<-NA;

timev<-NA; timevname<-NA;

bv<-X5;bvar<-"X5";bvname<-"X5";

colv<-X17.CS.NEW;colvname<-"X17.CS.NEW";

v.start<-NA; vname.start<-NA;

v.stop<-NA; vname.stop<-NA;

par1<-1;dec<-4;parm<-c(1,NA, NA, NA, 0);

if (!exists("pdfwd")) pdfwd<-6;

if (!exists("pdfht")) pdfht<-6;

##R package## pROC survivalROC plyr rms rmda Hmisc ##R package##;

pvformat<-function(p,dec) {

pp <- sprintf(paste("%.",dec,"f",sep=""),as.numeric(p))

if (is.matrix(p)) {pp<-matrix(pp, nrow=nrow(p)); colnames(pp)<-colnames(p);rownames(pp)<-rownames(p);}

lw <- paste("<",substr("0.00000000000",1,dec+1),"1",sep="");

pp[as.numeric(p)<(1/10^dec)]<-lw

return(pp)

}

numfmt<-function(p,dec) {

if (is.list(p)) p<-as.matrix(p)

pp <- sprintf(paste("%.",dec,"f",sep=""),as.numeric(p))

if (is.matrix(p)) {pp<-matrix(pp, nrow=nrow(p));colnames(pp)<-colnames(p);rownames(pp)<-rownames(p);}

pp[as.numeric(p)>10000000]<- "inf."

pp[is.na(p) | gsub(" ","",p)==""]<- ""

pp[p=="-Inf"]<-"-Inf"

pp[p=="Inf"]<-"Inf"

return(pp)

}

rocplot<-function(x,xb) {

xlm<-c(0,1);

x1 <- 1-x$specificities; y1<-x$sensitivities;

if (x$percent) {x1<-x1/100; y1<-y1/100;}

plot(x1, y1, xlim=c(0, 1), ylim=c(0, 1), xlab="1 - Specificity", ylab="Sensitivity",

asp=0.9, mar=c(4, 4, 2, 2)+.1, mgp=c(2.5, 1, 0), col=par("col"),

lty=par("lty"), lwd=2, type="l", main=paste(xb, "\nAUC =", round(x$auc,3)));

abline(a=0,b=1,col="darkgrey",lty=1,lwd=1);

}

rocplot2<-function(x,xb,mainb) {

n=length(x); xlm<-c(0,1); ccol<-(1:n)

x1 <- 1-x[[1]]$specificities; y1<-x[[1]]$sensitivities; auc<-x[[1]]$auc

if (x[[1]]$percent) {x1<-x1/100; y1<-y1/100;}

if (n==1) {mb<-paste(xb[1],"\nAUC =", round(auc,3));

} else {mb<-mainb;}

plot(x1, y1, xlim=xlm, ylim=xlm,

xlab="1 - Specificity", ylab="Sensitivity",

asp=0.9, mar=c(4, 4, 2, 2)+.1, mgp=c(2.5, 1, 0),

col=1, lty=1, lwd=2, type="l", main=mb);

abline(a=0,b=1,col="darkgrey",lty=1,lwd=1);

for (k in (2:n)) {

auc<-c(auc,x[[k]]$auc)

par(new=T)

xk <- 1-x[[k]]$specificities; yk<-x[[k]]$sensitivities

if (x[[k]]$percent) {xk<-xk/100; yk<-yk/100;}

plot(xk, yk, xlim=xlm, ylim=xlm, xlab="", ylab="",

asp=0.9, mar=c(4, 4, 2, 2)+.1, mgp=c(2.5, 1, 0),

col=k, lty=1, lwd=2, type="l", main="");

}

if (n>1) {

mlen<-min(max(nchar(xb)),15);

mspace<-paste(rep(" ",mlen),collapse="")

xb1<-substr(paste(xb,mspace),1,mlen)

tauc<-paste(xb1,pvformat(auc,3),sep=": ")

ord<-order(auc,decreasing=TRUE)

tauc<-tauc[ord]; clr<-(1:n)[ord]

legend(0.6,0.4,tauc,title="AUC",col=clr,lty=1,lwd=1,bty="n")

}

}

diagss<-function(d,x,xb,xlb,ylb,pngf) {

cmp<- !is.na(d) & !is.na(x); d<-d[cmp]; x<-x[cmp]

d0<-d; d<-2-d; ndL <- length(levels(factor(d))); nxL <- length(levels(factor(x)));

if (ndL!=2 | nxL<2) return (c(xb,rep(" ",6)))

lr <- rep(NA,nxL)

if (nxL==2) {x<-2-x; xlb<-c(xlb[2],xlb[1])}

t0<-table(x,d); ctot<-apply(t0,2,sum); rtot<-apply(t0,1,sum)

for (i in (1:nrow(t0))) lr[i] <- (t0[i,1]/ctot[1]) / (t0[i,2]/ctot[2])

tt <- rbind(c(xb, " "," "," "),cbind(xlb,t0,format(round(lr,dec),nsmall=dec)))

if (nxL==2) {

sen <- t0[1,1]/ctot[1]; spe<- t0[2,2]/ctot[2]

ppv <- t0[1,1]/rtot[1]; npv<- t0[2,2]/rtot[2]

acc<-(t0[1,1]+t0[2,2])/(ctot[1]+ctot[2])

plr<-sen/(1-spe); nlr<-(1-sen)/spe; nnd<-1/(sen+spe-1); dor<-plr/nlr

tt<-cbind(tt,rbind(format(round(c(sen,spe,ppv,npv,acc,plr,nlr,dor,nnd),dec),nsmall=dec),matrix(" ",nrow=nxL,ncol=9)))

} else {

tmp.glm<-glm(d0~factor(x),family=binomial(link="logit"))

roc0<-roc(d0,predict(tmp.glm))

a.tss<-roc2xls(roc0,d0)

colnames(a.tss)<-c("threshold","specificity","sensitivity","accuracy","positive-LLR","negative-LLR","diagnose-OR","N-for-diagnose","postive-pv","negative-pv","a","b","c","d")

bb<-format(round(a.tss[which.max(a.tss[,2]+a.tss[,3]),c(3,2,9,10,4,5,6,7,8)],dec),nsmall=dec)

xlsfname<-paste(pngf,"_roc.xls",sep="")

write.table(a.tss,file=xlsfname,row.names=FALSE,col.names=TRUE,sep="\t",append=FALSE,quote=FALSE)

png(paste(ofname,"_raw.png",sep="")); rocplot(roc0,xb); dev.off()

pdf(paste(ofname,"_raw.pdf",sep=""), width=pdfwd, height=pdfht, family="Helvetica"); rocplot(roc0,xb); dev.off()

tt<-cbind(tt,rbind(bb,matrix(" ",nrow=(nrow(tt)-1),ncol=9)))

}

return(tt)

}

roc2xls<-function(roc0,d) {

a.tss<-t(coords(roc0,"all",ret=c("threshold", "specificity", "sensitivity"), transpose=TRUE))

bdac<-table(d); t.bd<-bdac[1]; t.ac<-bdac[2]

t.a<-a.tss[,3]*t.ac; t.c<-t.ac-t.a; t.d<-a.tss[,2]*t.bd; t.b<-t.bd-t.d;

acc<-(t.a+t.d)/(t.ac+t.bd)

plr<-(t.a/(t.a+t.c))/(t.b/(t.b+t.d))

nlr<-(t.c/(t.a+t.c))/(t.d/(t.b+t.d))

dor<-plr/nlr

nnd<-1/(t.a/(t.a+t.c)-t.b/(t.b+t.d))

ppv<-t.a/(t.a+t.b)

npv<-t.d/(t.c+t.d)

a.tss<-cbind(a.tss,acc,plr,nlr,dor,nnd,ppv,npv,t.a,t.b,t.c,t.d)

return(a.tss)

}

glm2formula<-function(mdl) {

coe<-summary(mdl)$coefficients;

tmp<-rownames(coe);

tmp<-gsub(")","=",tmp)

tmp[substr(tmp,1,7)=="factor("]<-paste(substr(tmp[substr(tmp,1,7)=="factor("],7,99),")",sep="")

tmp<-paste("*",tmp,sep="")

tmp[1]<-"";

tme<-gsub(" ","",numfmt(coe[,1],5));

tme[as.numeric(tme)>0]<-paste("+",tme[as.numeric(tme)>0],sep="")

tme<-paste(tme,tmp,sep="",collapse=" ")

if (substr(tme,1,1)=="+") tme<-substr(tme,2,999);

return(paste("logit(",colvname,") = ",tme,sep=""))

}

diagrocx1<-function(d,x,xb,pngfname) {

cmp<- !is.na(d) & !is.na(x); d<-d[cmp]; x<-x[cmp]

ndL <- length(levels(factor(d)));

tt<-c(xb,format(c(sum(d),length(d)-sum(d)),nsmall=0))

if ((min(x)==max(x)) | ndL!=2) return (c(tt,rep(" ",3)))

roc0<-roc(d,x)

a.tss<-roc2xls(roc0,d)

colnames(a.tss)<-c("threshold","specificity","sensitivity","accuracy","positive-LLR","negative-LLR","diagnose-OR","N-for-diagnose","postive-pv","negative-pv","a","b","c","d")

b.tss<-a.tss[which.max(a.tss[,2]+a.tss[,3]),]

xlsfname<-paste(pngfname,"_roc.xls",sep="")

write.table(a.tss,file=xlsfname,row.names=FALSE,col.names=TRUE,sep="\t",append=FALSE,quote=FALSE)

png(paste(pngfname,"_raw.png",sep="")); rocplot(roc0,xb); dev.off()

pdf(paste(pngfname,"_raw.pdf",sep=""), width=pdfwd, height=pdfht, family="Helvetica"); rocplot(roc0,xb); dev.off()

roc1<-try(roc(d,x,smooth=bt.smooth,ci=TRUE,boot.n=bt.times,ci.alpha=0.95))

if (length(roc1)>2) {

if (bt.smooth) {

png(paste(pngfname,".png",sep="")); rocplot(roc1,xb); dev.off()

pdf(paste(pngfname,".pdf",sep=""), width=pdfwd, height=pdfht, family="Helvetica"); rocplot(roc1,xb); dev.off()

sens.ci<-try(ci.se(roc1,specificities=seq(0,1,0.05)))

if (length(sens.ci)>1) {

png(paste(pngfname,"_ci.png",sep=""))

plot(roc1,grid=TRUE,legacy.axes=TRUE,main=paste(xb, "\nAUC = ",round(roc1$auc,3)))

plot(sens.ci,type="shape",col="lightblue")

plot(sens.ci,type="bars")

dev.off()

pdf(paste(pngfname,"_ci.pdf",sep=""), width=pdfwd, height=pdfht, family="Helvetica");

plot(roc1,grid=TRUE,legacy.axes=TRUE,main=paste(xb, "\nAUC = ",round(roc1$auc,3)))

plot(sens.ci,type="shape",col="lightblue")

plot(sens.ci,type="bars")

dev.off()

}

}

aucci<-c(numfmt(c(roc1$auc,roc1$ci[c(1,3)],b.tss[1:10]),dec),b.tss[11:14])

} else {aucci<-rep(NA,17);}

return(list(c(tt,aucci),roc0))

}

diagrocx2<-function(d,x1,x1b,x2,x2b,pngfname,k) {

cmp<- !is.na(d) & !is.na(x1) & !is.na(x2); d<-d[cmp];x1<-x1[cmp];x2<-x2[cmp]

ndL <- length(levels(factor(d)));

tt1<-c(x1b,format(c(sum(d),length(d)-sum(d)),nsmall=0))

tt2<-c(x2b,format(c(sum(d),length(d)-sum(d)),nsmall=0))

if (ndL!=2) return (rbind(c(tt1,rep(" ",3)),c(tt2,rep(" ",3))))

tt<-c("Test/model","D+","D-","AUC","AUC.low","AUC.upp");

cnm<-c("threshold", "specificity", "sensitivity","accuracy","positive-LLR","negative-LLR")

cnm<-c(cnm,"diagnose-OR","N-for-diagnose","postive-pv","negative-pv","a","b","c","d")

tt<-c(tt,cnm)

if (min(x1)!=max(x1)) {

roc0<-roc(d,x1)

a.tss1<-roc2xls(roc0,d)

colnames(a.tss1)<-cnm

b.tss<-a.tss1[which.max(a.tss1[,2]+a.tss1[,3]),]

png(paste(pngfname,"_raw1.png",sep="")); rocplot(roc0,x1b); dev.off()

pdf(paste(pngfname,"_raw1.pdf",sep=""), width=pdfwd, height=pdfht, family="Helvetica"); rocplot(roc0,x1b); dev.off()

roc1<-try(roc(d,x1,smooth=bt.smooth,ci=TRUE,boot.n=bt.times,ci.alpha=0.95))

if (length(roc1)>2) {

if (bt.smooth) {

png(paste(pngfname,"_1.png",sep="")); rocplot(roc1,x1b); dev.off()

pdf(paste(pngfname,"_1.pdf",sep=""), width=pdfwd, height=pdfht, family="Helvetica"); rocplot(roc1,x1b); dev.off()

sen1.ci<-try(ci.se(roc1,specificities=seq(0,1,0.05)))

if (length(sen1.ci)>1) {

png(paste(pngfname,"_ci1.png",sep=""))

plot(roc1,xlim=c(1.0,0.0),grid=TRUE,legacy.axes=TRUE,main=paste(x1b, "\n AUC = ",round(roc1$auc,3)))

plot(sen1.ci,type="shape",col="lightblue")

plot(sen1.ci,type="bars")

dev.off()

pdf(paste(pngfname,"_ci1.pdf",sep=""), width=pdfwd, height=pdfht, family="Helvetica");

plot(roc1,xlim=c(1.0,0.0),grid=TRUE,legacy.axes=TRUE,main=paste(x1b, "\n AUC = ",round(roc1$auc,3)))

plot(sen1.ci,type="shape",col="lightblue")

plot(sen1.ci,type="bars")

dev.off()

}

}

auc1<-c(tt1,numfmt(c(roc1$auc,roc1$ci[c(1,3)],b.tss[1:10]),dec),b.tss[11:14])

} else {auc1<-rep(NA,length(tt));}

tt1=cbind(tt,auc1)

}

if (min(x2)!=max(x2)) {

roc02<-roc(d,x2)

a.tss2<-roc2xls(roc02,d)

colnames(a.tss2)<-cnm

b.tss<-a.tss2[which.max(a.tss2[,2]+a.tss2[,3]),]

png(paste(pngfname,"_raw2.png",sep="")); rocplot(roc02,x2b); dev.off()

pdf(paste(pngfname,"_raw2.pdf",sep=""), width=pdfwd, height=pdfht, family="Helvetica"); rocplot(roc02,x2b); dev.off()

roc2<-try(roc(d,x2,smooth=bt.smooth,ci=TRUE,boot.n=bt.times,ci.alpha=0.95))

if (length(roc2)>2) {

if (bt.smooth) {

png(paste(pngfname,"_2.png",sep="")); rocplot(roc2,x2b); dev.off()

pdf(paste(pngfname,"_2.pdf",sep=""), width=pdfwd, height=pdfht, family="Helvetica");rocplot(roc2,x2b); dev.off()

sen2.ci<-try(ci.se(roc2,specificities=seq(0,1,0.05)))

if (length(sen2.ci)>1) {

png(paste(pngfname,"_ci2.png",sep=""))

plot.roc(roc2,xlim=c(1.0,0.0),grid=TRUE,legacy.axes=TRUE,main=paste(x2b, "\n AUC = ",round(roc2$auc,3)))

plot(sen2.ci,type="shape",col="lightblue")

plot(sen2.ci,type="bars")

dev.off()

pdf(paste(pngfname,"_ci2.pdf",sep=""), width=pdfwd, height=pdfht, family="Helvetica");

plot.roc(roc2,xlim=c(1.0,0.0),grid=TRUE,legacy.axes=TRUE,main=paste(x2b, "\n AUC = ",round(roc2$auc,3)))

plot(sen2.ci,type="shape",col="lightblue")

plot(sen2.ci,type="bars")

dev.off()

}

}

auc2<-c(tt2,numfmt(c(roc2$auc,roc2$ci[c(1,3)],b.tss[1:10]),dec),b.tss[11:14])

} else {auc2<-rep(NA,length(tt));}

tt1=cbind(tt1,auc2)

}

if ((min(x1)!=max(x1)) & (min(x2)!=max(x2))) {

if (nrow(a.tss1)==nrow(a.tss2)) {

a.tss<-cbind(a.tss1,a.tss2)

colnames(a.tss)<-c(paste(colnames(a.tss1),"1",sep="."), paste(colnames(a.tss2),"2",sep="."))

write.table(a.tss,file=paste(pngfname,"_roc.xls",sep=""),row.names=FALSE, col.names=TRUE, sep="\t",append=FALSE,quote=FALSE)

} else {

write.table(a.tss1,file=paste(pngfname,"_roc1.xls",sep=""),row.names=FALSE, col.names=TRUE, sep="\t",append=FALSE,quote=FALSE)

write.table(a.tss2,file=paste(pngfname,"_roc2.xls",sep=""),row.names=FALSE, col.names=TRUE, sep="\t",append=FALSE,quote=FALSE)

}

if (nbg>1) {tmp.mb<-paste(bvb,bv.lb[k],sep=": ");} else {tmp.mb<-paste("Compare 2 models");}

png(paste(pngfname,"_2raw.png",sep="")); rocplot2(list(roc0,roc02),c(x1b,x2b),tmp.mb); dev.off()

pdf(paste(pngfname,"_2raw.pdf",sep=""), width=pdfwd, height=pdfht, family="Helvetica");

rocplot2(list(roc0,roc02),c(x1b,x2b),tmp.mb); dev.off()

if (length(roc1)>2 & length(roc2)>2) {

p<-roc.test(roc1,roc2,reuse.auc=FALSE,boot.n=bt.times)$p.value

ccp<-c("P(compare)",rep(" ",2),format(round(p,dec),nsmall=dec))

ccp<-c(ccp,rep(" ",length(tt)-length(ccp)))

tt1=cbind(tt1,ccp)

if (bt.times>0) {

png(paste(pngfname,"_2smooth.png",sep="")); rocplot2(list(roc1,roc2),c(x1b,x2b),tmp.mb); dev.off()

pdf(paste(pngfname,"_2smooth.pdf",sep=""), width=pdfwd, height=pdfht, family="Helvetica");

rocplot2(list(roc1,roc2),c(x1b,x2b),tmp.mb); dev.off()

}

}

}

if (!is.matrix(tt1)) {

return(rep(" ",length(tt)));

} else {

if (ncol(tt1)>1) {return(tt1[,-1])} else {return(rep(" ",length(tt)))}

}

}

plotnomogram <- function(tmp.nom) {

tmp.nom.x<-tmp.nom[length(tmp.nom)][[1]]$x

x.mid<-(max(tmp.nom.x)+min(tmp.nom.x))/2

x.mid.dis<-abs(tmp.nom.x-x.mid)

x.setblank<-(1:length(tmp.nom.x))[-c(1,which(x.mid.dis==min(x.mid.dis)),length(tmp.nom.x))]

tmp.nom[length(tmp.nom)][[1]]$fat[x.setblank]<-""

lbl.nchar<-max(nchar(c(names(tmp.nom),"linear.predictor")))

lblfac<-0.2

if (lbl.nchar>20) lblfac<-0.3

if (lbl.nchar>30) lblfac<-0.4

plot(tmp.nom,xfrac=lblfac);

}

mdl2oddsratio<-function(mdlobj, rr="OR") {

tmps <- summary(mdlobj)$coefficients

tmpc <- cbind(tmps[,1],tmps[,1]-1.96*tmps[,2],tmps[,1]+1.96*tmps[,2])

tmpor<- cbind(exp(tmpc),tmps[,4])

colnames(tmpor) <- c(rr,"Low 95%CI","High 95%CI", "P value")

rownames(tmpor) <- rownames(tmps)

return(tmpor)

}

mat2htmltable<-function(mat, rcname=FALSE) {

if (rcname) mat<-cbind(c("",rownames(mat)),rbind(colnames(mat),mat))

t1<- apply(mat,1,function(z) paste(z,collapse="</td><td>"))

t2<- paste("<tr><td>",t1,"</td></tr>")

return(paste(t2,collapse=" "))

}

vlabelN<-(substr(vlabel,1,1)==" ");

vlabelZ<-vlabel[vlabelN];vlabelV<-vlabel[!vlabelN]

vnameV<-vname[!vlabelN];vnameZ<-vname[vlabelN]

if (is.na(parm[1])) parm[1]<-0

bt.smooth<-(parm[1]==1); bt.times<-500;

if (is.na(parm[2])) {

study.type <- "cohort";

} else {

if (parm[2]>=1) parm[2] <- parm[2]/100

if (parm[2]>0) {study.type<-"case-control"; population.r <- parm[2];} else {study.type="cohort";}

}

if (is.na(avname[1]) & par1==3) par1==2;

if (!is.na(bvar)) {

bvb<-vlabelV[match(bvar,vnameV)]; if (is.na(bvb)) bvb<-bvar;

bv.lv<-levels(factor(bv));

bv.lb<-vlabelZ[match(paste(bvar,bv.lv,sep="."),vnameZ)]

bv.lb[is.na(bv.lb)]<-bv.lv[is.na(bv.lb)]

nbg<-length(bv.lv);

} else {bv<-rep(1,nrow(WD)); bv.lv<-rep(1,nrow(WD)); nbg<-1; bvb<-"ALL";}

nx=ncol(xv); xb<-vlabelV[match(xvname,vnameV)]; xb[is.na(xb)]<-xvname[is.na(xb)];

colv.lv<-levels(factor(colv))

yb<-vlabelV[match(colvname,vnameV)]; if (is.na(yb)) yb<-colvname;

ylb<-c("0","1")

if (length(colv.lv)==2 & colv.lv[1]==0 & colv.lv[2]==1) {

yv<-colv;

} else {

if (length(colv.lv)>=2) {

yv<-(colv==colv.lv[length(colv.lv)])*1; yv[is.na(colv)]<-NA;

ylb<-c("0",paste(colvname,"=",colv.lv[length(colv.lv)],sep=""))

} else {par1==0;}

}

ofname1<-ofname;

tmp.ss<-rep(NA,13); if (nbg>1) tmp.ss<-rep(NA,14)

tmp.roc<-rep(NA,20)

dec<-4; rocc<-list(NA)

sink(paste(ofname1,".txt",sep=""))

tmp.fml<-NULL

w.00<-""

tmp1<-c("Test","1","0","ROC area(AUC)","95%CI low","95%CI upp","Best threshold","Specificity","Sensitivity")

tmp1<-c(tmp1,"Accuracy","Positive-LR","Negative-LR","Diagnose-OR","N-for-diagnose","Postive-pv","Negative-pv","a","b","c","d")

for (k in (1:nbg)) {

ncxplot<-0

if (nbg>1) {

print(paste(bvb,"=",bv.lb[k]))

w.00<-c(w.00,"</br>", paste(bvb,"=",bv.lb[k]), "</br></br>")

}

if (par1==1) {

dcas <- list()

ndca <- 0

dcaname <- NULL

for (i in (1:nx)) {

x.tmp<-xv[bv==bv.lv[k],i]; y.tmp<-yv[bv==bv.lv[k]]

cmp<-(!is.na(x.tmp) & !is.na(y.tmp))

x.tmp<-x.tmp[cmp]; y.tmp<-y.tmp[cmp]; WDTMP <- as.data.frame(cbind(y.tmp,x.tmp))

singleChk.x<-(max(x.tmp)!=min(x.tmp))

if (length(levels(factor(y.tmp)))==2 & singleChk.x) {

pngf<-paste(ofname1,xvname[i],sep="_")

if (nbg>1) pngf<-paste(pngf,bv.lv[k],sep="_")

if (xlv[i]==0) {

tmp.xb<-xb[i]; if (nbg>1) tmp.xb<-paste(tmp.xb," (",bvb,"=",bv.lb[k],")",sep="")

tmp<-diagrocx1(y.tmp,x.tmp,tmp.xb,pngf)

tmp.roc<-rbind(tmp.roc,tmp[[1]])

ncxplot<-ncxplot+1

rocc[[ncxplot]]<-tmp[[2]]

if (ncxplot==1) {xxb<-tmp.xb;} else {xxb<-c(xxb,tmp.xb);}

ndca <- ndca + 1

dcas[[ndca]] <- decision_curve(y.tmp~x.tmp, data=WDTMP, study.design="cohort", policy="opt-in", bootstraps = 50)

write.table(dcas[[ndca]]$derived.data,file=paste(pngf,xvname[i],"dca.xls",sep="_"),col.names=TRUE,row.names=FALSE,sep="\t")

if (is.null(dcaname)) {dcaname <- xvname[i];} else {dcaname<-c(dcaname, xvname[i]);}

} else {

xi.lv<-levels(factor(x.tmp))

tmp.xlb<-vlabelZ[match(paste(xvname[i],xi.lv,sep="."),vnameZ)]

tmp.xlb[is.na(tmp.xlb)]<-xi.lv[is.na(tmp.xlb)]

tmp<-diagss(y.tmp,x.tmp,xb[i],tmp.xlb,ylb,pngf)

if (nbg>1) tmp<-cbind(bv.lb[k],tmp)

tmp.ss<-rbind(tmp.ss,tmp)

}

}

rm(WDTMP)

}

if (ncxplot>1) {

pngf<-ofname1; if (nbg>1) pngf<-paste(pngf,bv.lv[k],sep="_")

if (nbg>1) {tmp.mb<-paste(bvb,bv.lb[k],sep=": ");} else {tmp.mb<-paste("ROC curve for", yb);}

png(paste(pngf,"_rocs.png",sep=""),width=640,height=640); rocplot2(rocc,xxb,tmp.mb); dev.off()

pdf(paste(pngf,"_rocs.pdf",sep=""),width=pdfwd, height=pdfht, family="Helvetica");rocplot2(rocc,xxb,tmp.mb); dev.off()

}

if (is.matrix(tmp.ss)) {

tmp0<-c("Test","1","0","Likelihood ratio(LR)","Sensitivity","Specificity","Positive pv","Negative pv","Accuracy","Positive-LR","Negative-LR","Diagnosis OR","Number for diagnose")

if (nbg>1) tmp0<-c(bvb,tmp0)

tmp.ss<-rbind(tmp0,tmp.ss[-1,])

w.00<-c(w.00,"</br>Sensitivity and Specificity for diagnositic test</br><table border=3>",mat2htmltable(tmp.ss),"</table>")

}

if (is.matrix(tmp.roc)) {

tmp.roc<-rbind(tmp1,tmp.roc[-1,])

tmp.roc1<-tmp.roc[,(1:9)]

tmp.roc2<-tmp.roc[,-(2:6)]

w.00<-c(w.00,"</br>ROC analysis for continuous predictor</br><table border=3>",mat2htmltable(tmp.roc1),"</table>")

w.00<-c(w.00,"</br>Best threshold analysis</br><table border=3>",mat2htmltable(tmp.roc2),"</table>")

}

if (ndca>0) {

png(paste(pngf,"_dca.png",sep=""),width=960,height=840);

if (study.type=="cohort") {

plot_decision_curve(dcas, curve.names = dcaname, col = (2:(ndca+1)), confidence.intervals = FALSE)

} else {

plot_decision_curve(dcas, curve.names = dcaname, col = (2:(ndca+1)), confidence.intervals = FALSE,

study.design = "case-control", population.prevalence = population.r)

}

dev.off()

pdf(paste(pngf,"_dca.pdf",sep=""),width=pdfwd*1.5, height=pdfht*1.5, family="Helvetica");

if (study.type=="cohort") {

plot_decision_curve(dcas, curve.names = dcaname, col = (2:(ndca+1)), confidence.intervals = FALSE)

} else {

plot_decision_curve(dcas, curve.names = dcaname, col = (2:(ndca+1)), confidence.intervals = FALSE,

study.design = "case-control", population.prevalence = population.r)

}

dev.off()

}

}

if (par1==2) {

WD0<-cbind(yv,xv); colnames(WD0)<-c(colvname,xvname);

if (nbg>1) WD0<-WD0[bv==bv.lv[k],]

cmp<-(apply(is.na(WD0),1,sum)==0)

WD0<-as.data.frame(WD0[cmp,])

if (length(levels(factor(WD0[,1])))==2) {

xv1.1<-xvname

xv1.1[xlv>2]<-paste("factor(",xv1.1[xlv>2],")",sep="")

singleChk<- apply(cbind(WD0[,xvname],1),2,function(x) return(max(x,na.rm=TRUE)-min(x,na.rm=TRUE)!=0))

fml.1<-paste(colvname,"~",paste(xv1.1[singleChk],collapse="+"))

tmp.glm<-try(glm(formula(fml.1),family=binomial(link="logit"),data=WD0))

pngf<-paste(ofname1,"model",sep="_")

if (nbg>1) pngf<-paste(pngf,bv.lv[k],sep="_")

if (substr(tmp.glm[[1]][1],1,5)!="Error") {

print(summary(tmp.glm)); print(mdl2oddsratio(tmp.glm))

tmp.lrm<-try(lrm(formula(fml.1),data=WD0))

if (substr(tmp.lrm[[1]][1],1,5)!="Error") {

tmp.dst<-datadist(WD0)

options(datadist='tmp.dst')

tmp.nom<-nomogram(tmp.lrm, fun=function(x)1/(1+exp(-x)),funlabel=yb)

print("Nomogram"); print(tmp.nom)

png(paste(pngf,"_nom.png",sep=""),width=960,height=840); plotnomogram(tmp.nom); dev.off()

pdf(paste(pngf,"_nom.pdf",sep=""),width=pdfwd*1.5, height=pdfht*1.5, family="Helvetica");plotnomogram(tmp.nom); dev.off()

}

x.tmp<-predict(tmp.glm)

y.tmp<-WD0[,1]

tmp.xb<-"Model";

tmpi<-glm2formula(tmp.glm)

if (nbg>1) {

tmp.xb<-paste(tmp.xb," (",bvb,"=",bv.lb[k],")",sep="")

tmpi<-paste("</br>",bvb,"=",bv.lb[k],":</br>",tmpi)

}

tmp.fml<-c(tmp.fml,tmpi)

tmp<-diagrocx1(y.tmp,x.tmp,tmp.xb,pngf)

tmp.roc<-cbind(tmp.roc,tmp[[1]])

dca0 <- decision_curve(formula(fml.1), data=WD0, study.design="cohort", policy="opt-in", bootstraps = 50)

png(paste(pngf,"_dca.png",sep=""),width=960,height=840);

if (study.type=="cohort") {

plot_decision_curve(dca0, curve.names="Model", confidence.intervals = FALSE, col="red")

} else {

plot_decision_curve(dca0, curve.names="Model", confidence.intervals = FALSE, col="red",

study.design = "case-control", population.prevalence = population.r)

}

dev.off()

pdf(paste(pngf,"_dca.pdf",sep=""),width=pdfwd*1.5, height=pdfht*1.5, family="Helvetica");

if (study.type=="cohort") {

plot_decision_curve(dca0, curve.names="Model", confidence.intervals = FALSE, col="red")

} else {

plot_decision_curve(dca0, curve.names="Model", confidence.intervals = FALSE, col="red",

study.design = "case-control", population.prevalence = population.r)

}

dev.off()

write.table(dca0$derived.data,file=paste(pngf,"_dca.xls",sep=""),col.names=TRUE,row.names=FALSE,sep="\t")

} else {tmp.roc<-"Model error!";}

rm(WD0)

}

if (is.matrix(tmp.roc)) {

tmp.roc<-cbind(tmp1,tmp.roc[,-1])

} else {w.00<-c(w.00,"</br>",tmp.roc);}

}

if (par1==3) {

WD0<-cbind(yv,xv,av); colnames(WD0)<-c(colvname,xvname,avname)

if (nbg>1) WD0<-WD0[bv==bv.lv[k],]

cmp<-(apply(is.na(WD0),1,sum)==0)

WD0<-as.data.frame(WD0[cmp,])

if (length(levels(factor(WD0[,1])))==2) {

xv1<-xvname

xv1[xlv!=0]<-paste("factor(",xv1[xlv!=0],")",sep="")

singleChk.xv1 <- apply(cbind(WD0[,xvname],1),2,function(x) return(max(x)-min(x)!=0))

xv1<- xv1[singleChk.xv1];

fml<-paste(colvname,"~",paste(xv1,collapse="+"))

tmp.glm<-try(glm(formula(fml),family=binomial(link="logit"),data=WD0))

if (substr(tmp.glm[[1]][1],1,5)!="Error") {

or.tb1<- numfmt(mdl2oddsratio(tmp.glm),4)

print(summary(tmp.glm)); print(or.tb1)

x.tmp<-predict(tmp.glm)

y.tmp<-WD0[,1]

av1<-avname

av1[alv!=0]<-paste("factor(",av1[alv!=0],")",sep="")

singleChk.av1 <- apply(cbind(WD0[,avname],1),2,function(x) return(max(x)-min(x)!=0))

av1<- av1[singleChk.av1];

fml2<-paste(colvname,"~",paste(av1,collapse="+"))

tmp.glm2<-try(glm(formula(fml2),family=binomial(link="logit"),data=WD0))

if (substr(tmp.glm2[[1]][1],1,5)!="Error") {

or.tb2<- numfmt(mdl2oddsratio(tmp.glm2),4)

print(summary(tmp.glm2)); print(or.tb2)

x.tmp2<-predict(tmp.glm2)

pngf<-paste(ofname1,"model2",sep="_")

if (nbg>1) pngf<-paste(pngf,bv.lv[k],sep="_")

tmp.x1b<-"Model1"; if (nbg>1) tmp.x1b<-paste(tmp.x1b,"(",bvb,"=",bv.lb[k],")",sep="")

tmp.x2b<-"Model2"; if (nbg>1) tmp.x2b<-paste(tmp.x2b,"(",bvb,"=",bv.lb[k],")",sep="")

tmp.fml<-glm2formula(tmp.glm)

tmp.fml2<-glm2formula(tmp.glm2)

tmp.roc<-cbind(tmp.roc,diagrocx2(y.tmp,x.tmp,tmp.x1b,x.tmp2,tmp.x2b,pngf,k))

impr<-improveProb(x.tmp>=as.numeric(tmp.roc[7,2]), x.tmp2>=as.numeric(tmp.roc[7,3]), y.tmp)

print(impr)

w.nrp<-c("Increase for events (1)", "Increase for non-events (2)", "Decrease for events (3)", "Decrease for non-events (4)")

w.nrp<-cbind(w.nrp, numfmt(unlist(impr[c("pup.ev","pup.ne","pdown.ev","pdown.ne")]), dec))

nri<-matrix(unlist(impr[c("nri", "nri.ev", "nri.ne", "idi", "se.nri", "se.nri.ev", "se.nri.ne", "se.idi")]),ncol=2)

nri<-cbind(nri, nri[,1]/nri[,2])

nri<-cbind(nri, 2-2*pnorm(abs(nri[,3])))

nri<-cbind(nri, nri[,1]-1.96*nri[,2], nri[,1]+1.96*nri[,2])

w.nri<-numfmt(nri,dec)

w.nri[,4]<-pvformat(nri[,4],4)

w.nri<-rbind(c("Estimate","SE","Z","P value","95% Lower","95% Upper"), w.nri)

w.nri<-cbind(c("","NRI (1-3+4-2)","NRI for events (1-3)","NRI for non-events (4-2)","IDI"),w.nri)

dca1 <- decision_curve(formula(fml), data=WD0, study.design="cohort", policy="opt-in", bootstraps = 50)

dca2 <- decision_curve(formula(fml2), data=WD0, study.design="cohort", policy="opt-in", bootstraps = 50)

png(paste(pngf,"_dca.png",sep=""),width=960,height=840);

if (study.type=="cohort") {

plot_decision_curve(list(dca1, dca2), curve.names = c("Model 1", "Model 2"), col = c("blue", "red"), confidence.intervals = FALSE)

} else {

plot_decision_curve(list(dca1, dca2), curve.names = c("Model 1", "Model 2"), col = c("blue", "red"), confidence.intervals = FALSE,

study.design = "case-control", population.prevalence = population.r)

}

dev.off()

pdf(paste(pngf,"_dca.pdf",sep=""),width=pdfwd*1.5, height=pdfht*1.5, family="Helvetica");

if (study.type=="cohort") {

plot_decision_curve(list(dca1, dca2), curve.names = c("Model 1", "Model 2"), col = c("blue", "red"), confidence.intervals = FALSE)

} else {

plot_decision_curve(list(dca1, dca2), curve.names = c("Model 1", "Model 2"), col = c("blue", "red"), confidence.intervals = FALSE,

study.design = "case-control", population.prevalence = population.r)

}

dev.off()

write.table(dca1$derived.data,file=paste(pngf,"_dca1.xls",sep=""),col.names=TRUE,row.names=FALSE,sep="\t")

write.table(dca2$derived.data,file=paste(pngf,"_dca2.xls",sep=""),col.names=TRUE,row.names=FALSE,sep="\t")

} else {tmp.roc<-"Model 2 error!";}

} else {tmp.roc<-"Model 1 error!";}

rm(WD0)

}

if (is.matrix(tmp.roc)) {

tmp.roc<-cbind(tmp1,tmp.roc[,-1])

w.00<-c(w.00,"</br>Predictive model:</br>","Model 1：",tmp.fml,"</br>","Model 2: ",tmp.fml2)

w.00<-c(w.00,"</br></br>Compare 2 predictive models (ROC Curve)</br><table border=3>",mat2htmltable(tmp.roc),"</table>")

w.00<-c(w.00,"</br></br>Net Reclassification Index, Integrated Discrimination Index")

w.00<-c(w.00,"</br><table border=3>",mat2htmltable(w.nrp),"</table>")

w.00<-c(w.00,"</br><table border=3>",mat2htmltable(w.nri),"</table>")

w.00<-c(w.00,"</br>Model 1</br><table border=3>",mat2htmltable(or.tb1,TRUE),"</table>")

w.00<-c(w.00,"</br>Model 2</br><table border=3>",mat2htmltable(or.tb2,TRUE),"</table>")

} else {w.00<-c(w.00,"</br>",tmp.roc);}

}

}

sink()

w<-c("<html><head>","<meta http-equiv=\"Content-Type\" content=\"text/html\" charset=\"gb2312\" /></head><body>")

w<-c(w,"<h2>",title,"</h2>")

w<-c(w,"</br>Outcome variable:", yb, "</br>")

if (par1==2) {

w <-c(w,"</br>Predictive model:</br>",tmp.fml)

tmp1<-c("Test items","1","0","ROC area(AUC)","95% Interval lower limit","95% Interval upper limit","Optimal threshold","Specificity","Sensitivity")

tmp1<-c(tmp1,"Accuracy","Positive-LR","Negative-LR","Diagnosis OR","Number for diagnose","Positive pv","Negative pv","a","b","c","d");

tmp.roc<-cbind(tmp1,tmp.roc[,-1])

w<-c(w,"</br></br>Model ROC curve and best threshold analysis</br><table border=3>",mat2htmltable(tmp.roc),"</table>")

} else {

w<-c(w,w.00)

w<-c(w,"</br>Sensitivity; Specificity; Positive pv; Negative pv; Accuracy; Positive-LR; Negative-LR; Diagnosis OR; Number for diagnose </br>")

}

if (bt.smooth) w<-c(w,paste("</br>AUC confidence interval and significance test using non-parametric repeated sampling method (Bootstrap resampling times=",bt.times,")"))

w<-c(w,"</br></br> The optimal threshold is taken as the cut-off value with the maximum sensitivity + specificity. The sensitivity specificity corresponding to each cut-off point is saved in the ROC output file (.xls) ")

w<-c(w,"</body></html>")

fileConn<-file(paste(ofname1,".htm",sep="")); writeLines(w, fileConn)
